# Cognitive-Affective Inconsistency and Ambivalence: Impact on the Overall Attitude–Behavior Relationship

**DOI:** 10.1177/0146167220945900

**Published:** 2020-08-04

**Authors:** Mark Conner, Sarah Wilding, Frenk van Harreveld, Jonas Dalege

**Affiliations:** 1 University of Leeds, UK; 2 University of Amsterdam, The Netherlands

**Keywords:** attitudes, overall attitude, cognitive-affective inconsistency, cognitive-affective ambivalence, attitude–behavior relationship

## Abstract

This research explored whether overall attitude is a stronger predictor of behavior when underlying cognitive-affective inconsistency or ambivalence is low versus high. Across three prospective studies in different behaviors and populations (Study 1: eating a low-fat diet, *N* = 136 adults, eating five fruit and vegetables per day, *N* = 135 adults; Study 2: smoking initiation, *N* = 4,933 adolescents; and Study 3: physical activity, *N* = 909 adults) we tested cognitive-affective inconsistency and ambivalence individually and simultaneously as moderators of the overall attitude–behavior relationship. Across studies, more similar effects were observed for inconsistency compared with ambivalence (in both individual and simultaneous analyses). Meta-analysis across studies supported this conclusion with both cognitive-affective inconsistency and ambivalence being significant moderators when considered on their own, but only inconsistency being significant when tested simultaneously. The reported studies highlight the importance of cognitive-affective inconsistency as a determinant of the strength of overall attitude.

Attitudinal inconsistency exists when a component of a person’s attitude (i.e., affect, cognition, or behavior) is inconsistent with either their overall evaluation of the attitude object or other components of the same attitude (Maio et al., 2000). We here use “overall attitude” to describe the “overall evaluation” of the attitude object. Most research in this area has focused on whether such attitudinal inconsistency is a marker for attitude strength (Krosnick & Petty, 1995). In particular, research has tended to focus on whether attitudinal inconsistency makes overall attitudes weaker predictors of behavior, less stable over time, less resistant to change, and less impactful on information processing (Chaiken et al., 1995; Maio et al., 2000; Rosenberg, 1968). This article focuses on inconsistency between cognitive and affective evaluations (cognitive-affective inconsistency) and its moderating effect on the relationship between overall attitude and behavior. Importantly it also assesses whether any moderation effect is attenuated by controlling for a related construct, namely, cognitive-affective attitudinal ambivalence.

## Attitudinal Inconsistency

Various kinds of attitudinal inconsistency have been explored. A particular focus has been on evaluative-cognitive inconsistency. This refers to the extent to which a person’s overall evaluation on one hand and cognitions (or beliefs) about the same attitude object on the other, are inconsistent with one another (Rosenberg, 1968). Evaluative-cognitive inconsistency is usually operationalized as the absolute difference between two evaluations of the attitude object: one implied by overall evaluation of the object and another implied by cognitions about the attitude object (Chaiken & Baldwin, 1981; Rosenberg, 1968). Higher evaluative-cognitive inconsistency is inferred when the net evaluation of the overall evaluation is very different from that implied by a person’s cognitions about the attitude object. For example, having a positive *overall evaluation* of smoking, but *believing* smoking leads to negative outcomes such as cancer would represent high evaluative-cognitive inconsistency. High levels of this form of attitudinal inconsistency have been shown to be associated with low accessibility of attitude from memory, low attitude stability, and weak effects of attitudes on information processing (Chaiken et al., 1995). Other studies have explored evaluative-affective inconsistency (Chaiken et al., 1995; Erber et al., 1995). This refers to the extent to which overall evaluations of an attitude object are inconsistent with feelings (or emotions) about the same attitude object (Chaiken et al., 1995). For example, having a negative *overall evaluation* of smoking, but *enjoying* smoking would represent high evaluative-affective inconsistency.

Cognitive-affective inconsistency (or inter-component inconsistency as Maio et al., 2000 refer to it) has received only modest attention (Millar & Tesser, 1989). This kind of inconsistency refers to the extent to which a person’s cognitions (or beliefs) about an attitude object are inconsistent with his or her feelings (or emotions) about an attitude object (Maio et al., 2000). For example, *believing* smoking leads to negative outcomes such as cancer, but *enjoying* it nonetheless would represent high cognitive-affective inconsistency. Cognitive-affective inconsistency can be operationalized as the absolute difference between the evaluation implied by a person’s cognitions about the attitude object and their evaluation implied by their feelings about the attitude object (cp. Maio et al., 2000). Millar and Tesser (1989) showed that thought prior to forming an overall evaluation of an attitude object attenuated the attitude–behavior relationship only when cognitive-affective inconsistency was high and not when it was low. Sparks et al. (1992) showed that such a measure of cognitive-affective inconsistency (labeled ambivalence) moderated the attitude–behavioral intention relationship for one of two behaviors. For the behavior where the difference was significant, greater inconsistency was associated with weaker attitude–intention relationships. Schleicher et al. (2004) and also Visser and Coetzee (2005) showed that the relationship between job satisfaction and job performance was moderated by cognitive-affective inconsistency. In each of the three tests reported across studies, greater inconsistency was associated with a weaker relationship between job satisfaction and job performance.

Maio et al. (2000) reported that evaluative-cognitive, evaluative-affective, and cognitive-affective inconsistency were only modestly related to one another (*r* = .06–.42). Importantly, Maio et al. (2000) noted the conceptual overlap between measures of attitudinal inconsistency and measures of attitudinal ambivalence, although in their data the two were unrelated (*r* = −.25 to .06) (see Thompson et al., 1995).

## Attitudinal Ambivalence

A different kind of attitudinal inconsistency, and one that has been investigated more extensively in the literature, is attitudinal ambivalence. Definitions and measures of attitudinal ambivalence usually distinguish positive and negative evaluations of an attitude object and emphasize the similarity but also the intensity of the two evaluations (Conner & Sparks, 2002; Thompson et al., 1995). It is worth noting that this type of ambivalence is sometimes referred to as potential ambivalence to distinguish it from felt (or subjective) ambivalence. Felt ambivalence focuses on meta-cognitive awareness of the difference between positive and negative evaluations (Conner & Sparks, 2002) and is not the focus here. Potential attitudinal ambivalence increases as the positive and negative evaluations become more intense and similar. Similarity here would be the opposite of inconsistency. Thompson et al. (1995) suggest that positive (P) and negative (N) evaluations can be combined to produce a measure of ambivalence using the following equation (sometimes known as the Griffin formula):

$$\mathrm{Ambivalence}\;=\;\frac{\left(P+N\right)}{2}\;\_\;\mathrm{abs}\;(P\_N),$$

with the first component tapping intensity and the second similarity. As we discuss below, it is the similarity component that overlaps in measures of attitudinal inconsistency and ambivalence, and the intensity component that distinguishes these two constructs. These measures of ambivalence tend to focus on intra-component ambivalence and tap evaluative (i.e., positive and negative evaluations about an attitude object), cognitive (i.e., positive and negative cognitions about an attitude object), or affective (i.e., positive and negative affect about an attitude object) ambivalence. Unipolar attitude measures, such as Kaplan’s (1972) split semantic differential measure, are often used to tap these positive and negative components. Various studies support the idea that intra-component ambivalence is negatively related to the strength of an attitude. For example, several studies show ambivalent compared with non-ambivalent attitudes to be weaker predictors of behavior (e.g., Armitage & Conner, 2000). A smaller number of studies also show ambivalent attitudes to be less stable over time, more pliable, and more impactful on information processing (see Conner & Armitage, 2008 for a review).

Relatively few studies have examined inter-component attitudinal ambivalence (Maio et al., 2000), which is usually referred to as cognitive-affective ambivalence (Conner & Sparks, 2002). Two forms of cognitive-affective ambivalence can be distinguished and computed using the Griffin formula: pro-cognitive/con-affective ambivalence and con-cognitive/pro-affective ambivalence (Thompson et al., 1995). In the former, the individual’s cognitions are positively valenced toward the attitude-object, but their affect is negatively valenced toward the attitude object; in the latter, the individual’s cognitions are negatively valenced toward the attitude-object, but their affect is positively valenced toward the attitude object. When cognitive and affective evaluations are both tapped using unipolar attitude measures, the two types of inter-component cognitive-affective ambivalence and also both intra-component cognitive and intra-component affective ambivalence can be computed. However, this raises problems of how to best combine the two types of inter-component cognitive-affective ambivalence and also how to disconfound measures of inter-component and intra-component ambivalence (see Thompson et al., 1995). Unlike intra-component ambivalence, inter-component ambivalence can be tapped using bipolar measures of cognitive and affective evaluations. Using such bipolar evaluation measures also helps tackle both the above problems and is the approach used here. Such measures do not allow for intra-component ambivalence, although inter-component ambivalence can be computed when the cognitive and affective evaluations are oppositely valenced (i.e., different sides of the mid-point on the bipolar measure). In addition, with bipolar measures an individual will only have either pro-cognitive/con-affective *or* con-cognitive/pro-affective ambivalence, not both.

Work on intra-component attitudinal ambivalence has often considered it to be a measure of attitude strength (Thompson et al., 1995). A key prediction for any measure of attitude strength is that strong attitudes should be more likely to predict behavior than weak attitudes (Converse, 1995, p. 11; Krosnick & Petty, 1995, p. 3). Research on cognitive-affective ambivalence has investigated its impact on the prediction of behavior. Lavine et al. (1998) measured cognitive-affective ambivalence as having oppositely valenced affective and cognitive evaluations or not (i.e., no consideration of intensity). For respondents with oppositely valenced cognition and affect, affect exerted stronger influences on overall attitude and behavior. For respondents with similarly valenced cognition and affect, the two exerted equal influence on overall attitude and behavior. Although these results are insightful, the question of whether or not intra-component attitudinal ambivalence is an indication of a weak attitude in that it attenuates the overall attitude–behavior link remains unanswered.

In terms of why weak attitudes might be less predictive of behavior, Schwartz (1978) noted that an attitude assessed at one time point is unlikely to predict behavior at a later time point if it does not persist over the intervening time interval. Thus, at least part of the greater impact of strong attitudes on behavior may be attributable to strong attitudes being more likely to persist over time (Ajzen & Fishbein, 1980). However, other mechanisms may also play a role. For example, Fazio (1986, 1995) argued that attitudes influence our behavior in part by shaping our perceptions of the world (see Fabrigar et al., 2005 for a useful discussion of various ways that attitude structure could influence the attitude–behavior relationship), that is, the capacity of an attitude to predict behavior is partly dependent on the attitude’s ability to bias perceptions of the attitude object and the context in which the behavior is performed. Strong attitudes are assumed to be more readily accessible and thus more likely to produce these biasing effects. Alternatively, weak attitudes may be simply considered less relevant when deciding whether to perform a behavior. One or more of these factors may explain why low compared with high intra-component attitudinal ambivalence is associated with stronger predictions of behavior from attitude.

Similar arguments can be applied to inter-component cognitive-affective ambivalence. One contribution of this article is to offer a series of tests of whether cognitive-affective ambivalence does moderate the overall attitude to behavior relationship. Given that cognitive-affective inconsistency is also considered to be a component of attitude strength (Krosnick & Petty, 1995), it may also moderate the impact of overall attitude on behavior. Low inconsistency would equate to a strong overall attitude that should be more predictive of behavior. Providing a series of tests of this moderation effect is a second key contribution of this article. In addition, examining the moderating effects of cognitive-affective inconsistency plus ambivalence on the overall attitude to behavior relationship when controlling for one another (simultaneous test) is a third contribution of this article. Developing predictions in relation to this third contribution requires a more detailed consideration of the similarities and differences between the two.

## Similarities and Differences Between Cognitive-Affective Inconsistency and Ambivalence

**Figure 1.**
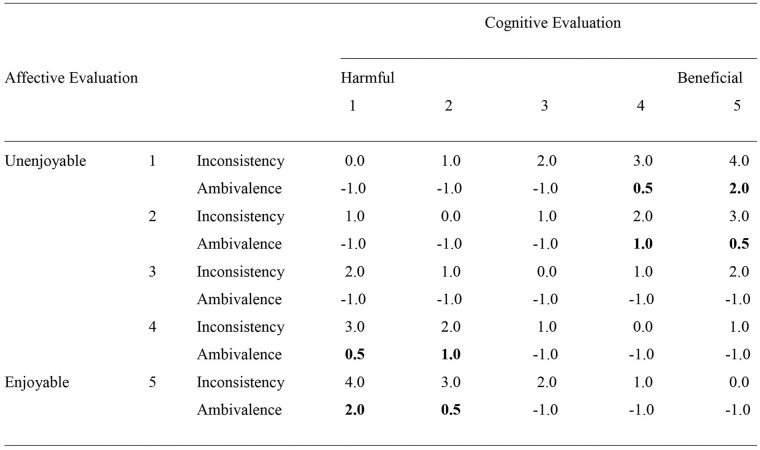
Scores for cognitive-affective inconsistency and cognitive-affective ambivalence at each level of cognitive and affective evaluation. *Note.* Cognitive-affective ambivalence is at a minimum (scored −1.0) except in the top right and bottom left quadrants of the matrix (marked in bold). Cognitive-affective inconsistency is at a minimum (scored 0.0) on the diagonal (top left to bottom right) where cognitive and affective evaluations are equal, but at a maximum (scored 4.0) in the top right and bottom left quadrants of the matrix.

Both cognitive-affective inconsistency and cognitive-affective ambivalence, when measured using bipolar measures of cognitive and affective evaluations, incorporate a component of the absolute difference between cognitive and affective evaluations in their measurement. On that basis we might expect them to be at least moderately positively correlated with one another (see Figure 1 for relationship between the two for different values of cognitive and affective evaluation). However, this difference score covers situations where cognitive and affective evaluations are both neutral (i.e., neither is valenced), only one is valenced, both are of the same valence, or one is the opposing valence to the other. It is only the latter case when cognitive-affective ambivalence occurs. In contrast, cognitive-affective inconsistency occurs whenever the two are not equal. In this sense cognitive-affective inconsistency can be seen as covering a broader range of combinations of cognitive and affective evaluations than cognitive-affective ambivalence. A further distinction between cognitive-affective inconsistency and ambivalence is that the latter incorporates a measure of the intensity of the cognitive and affective evaluations that is absent in the former. It is notable that Maio et al. (2000) reported that these two measures were unrelated in their data (*r* = .06). The present research offers further tests of the relationship between the two.

What implications do these similarities and differences have for predictions about the relative power of cognitive-affective inconsistency and ambivalence to moderate the overall attitude to behavior relationship? One mechanism through which cognitive-affective inconsistency and ambivalence might act as moderators is through an individual’s overall attitude being sometimes more aligned with their cognitive evaluations and sometimes more aligned with their affective evaluations (Abelson et al., 1982; Breckler & Wiggins, 1989; Esses et al., 1993). In line with this reasoning, Zhou et al. (2009) manipulated cognitive-affective ambivalence (which they labeled affective-cognitive consistency) and showed that this changed the extent to which attitude predicted behavior in a cognitive-focus condition, but not in an affective-focus condition. This might lead to overall attitude being less stable for those high versus low in cognitive-affective inconsistency or ambivalence. As noted above, such lower attitude stability might then drive lower attitude–behavior relationships. To the extent that lower stability of overall attitude is attributable to cognitive and affective evaluations being oppositely valenced and thus supporting different courses of action (i.e., approach vs. avoid), this might suggest that it is cognitive-affective ambivalence that is the stronger moderator. That is, when considered together, it is ambivalence and not inconsistency that moderates the overall attitude–behavior relationship. In contrast, the broader range of cognitive-affective combinations that inconsistency covers might suggest that it should be the stronger moderator of the overall attitude–behavior relationship (and ambivalence should not be significant when included with inconsistency). Support for cognitive-affective inconsistency being the stronger moderator would suggest that it is degree of inconsistency rather than opposing valence (or indeed intensity) of cognitive and affective evaluations that matters most in relation to attitude strength. For example, being neutral on one component and strongly positive/negative on the contrary may be a good indicator of a weak overall attitude that is tapped by an inconsistency measure, but not by an ambivalence measure. We test these different possibilities first by examining the moderating effects of cognitive-affective inconsistency or ambivalence on the overall attitude–behavior relationship (individual tests) and second by examining the moderating effects of each one when controlling for the moderating effects of the other (simultaneous tests).

In summary, the present research explored the moderating effects of cognitive-affective inconsistency and ambivalence on the relationship between overall attitude and subsequent behavior in real world settings. A novel focus was on the moderating effects of one when controlling for the other. Across studies we examined different health behaviors in different general populations with varying time gaps between measurement of the different components of attitude and later behavior. The overall effects across studies were assessed using meta-analysis.

## Study 1: Eating a Low-Fat Diet or Five Fruit and Vegetables Per Day

Study 1 focused on two dietary behaviors. Parts of the data were reported in Conner et al. (2002). A number of measures not reported here were also included (e.g., intentions; behavioral, normative, and control beliefs; overall intra-component attitudinal ambivalence). The full questionnaires (T1 and T2) are provided as an online appendix. The data are available here: https://osf.io/unz4s/?view_only=591a16ab02f04a49bc7947636ca6775b. The University of Leeds, UK ethical review committee approved the study.

### Method

#### Sample

The sample was recruited by advertisement placed in two local newspapers. Out of those who responded (*N* = 390), a total of 282 (72% response rate) fully completed and returned two questionnaires (T1 and T2, approximately one month apart) and were included in the analyses. Respondents were randomly allocated to either a completing questionnaires concerning eating a low-fat diet (*N* = 142) or concerning eating five portions of fruit and vegetables per day (*N* = 140). Respondents were paid £5 (approximately US$8) on receipt of the completed second questionnaire. There were 83 males and 199 females, mean age 54.2 years (*SD* = 17.6). After removing participants with any missing data on analyzed variables there were 136 for eating a low-fat diet and 135 for eating five portions of fruit and vegetables per day. The 271 analyzed respondents did not differ from the 11 excluded on baseline measures, *p* > .15.

#### Measures

Separate questionnaires were developed for the two behaviors (eating a low-fat diet; eating five portions of fruit and vegetables per day), although the design of each was very similar. Consistent with Crites et al. (1994) semantic differentials were classified into evaluative (overall attitude), cognitive, or affective. Overall attitude was assessed at T1 by three semantic differential items (e.g., “If I were to eat a low fat diet, it would be”: *“unfavorable-favorable,” “negative-positive,” “unsatisfactory-satisfactory”*; α = .80, .89 for low-fat diet and five portions a day, respectively). Cognitive evaluation was assessed at T1 by a single semantic differential item (e.g., “If I were to eat a low fat diet, it would be”: *“harmful-beneficial”*). Affective evaluation was assessed at T1 by two semantic differential items (e.g., “If I were to eat a low fat diet, it would be”: *“unpleasant-pleasant,” “unenjoyable-enjoyable”; r* = .86, .79 for low-fat diet and five portions a day, respectively). Each item was scored 1 to 7 (higher scores indicate more positive evaluations).

Cognitive-affective inconsistency was computed as the absolute difference between cognitive and affective evaluation measures (range 0–6).

A measure of cognitive-affective ambivalence was computed where the valence of the bipolar cognitive and affective evaluation measures was different (i.e., where the mean score on one measure was above the mid-point of the scale and the other mean score was below the mid-point of the scale). For respondents who were pro-cognitive/con-affective (i.e., mean cognitive evaluation above the mid-point 4 *plus* mean affective evaluation below the mid-point 4) *or* con-cognitive/pro-affective (i.e., mean cognitive evaluation below the mid-point 4 *plus* mean affective evaluation above the mid-point 4) we computed an ambivalence score. Cognitive and affective evaluation scores were assigned values of 1 to 3 based on extremity, with higher scores indicating greater extremity. These values were then entered into the Griffin equation (Ambivalence = [C + A]/2 − abs[C − A], where C is the cognitive evaluation and A is the affective evaluation). Respondents who, based on their responses to the cognitive and affective evaluation measures, were classified as neither pro-cognitive/con-affective or con-cognitive/pro-affective were coded as non-ambivalent (coded −1.5 to reflect the lowest level of ambivalence possible based on the positive score at the most extreme [3] and the negative score at the mid-point [0] or vice versa in the Griffin equation). The computed cognitive-affective ambivalence score range between −1.5 and +3.

Behavior was assessed at T2 using food frequency questionnaires. For eating a low-fat diet this was tapped by the frequency of choosing a series of low-fat options (e.g., “In the past month how often did you use skimmed milk, not applicable, rarely or never, sometimes, often, usually or always,” scored 0–4 and summed) (Block et al., 2000). For eating five portions of fruit and vegetables per day this was tapped by the frequency of choosing nine fruits and vegetables (e.g., “In the past month how often did you eat bananas, rarely or never, 13 times per month, 1-2 times per week, 3-5 times per week, every day, 2 or more times a day,” scored 0.001, 0.0667, 0.214, 0.571, 1, and 2, respectively, and summed to indicate number of portions per day; Cade & Margetts, 1988).

#### Analyses

Analyses were performed separately for the two behaviors. The means and standard deviations of the measures and their intercorrelations were examined first. Moderated multiple regression analysis was then used to test our main hypothesis about the moderating effect of cognitive-affective inconsistency and ambivalence on the overall attitude–behavior relationship. Variables were entered as predictors in a series of steps based on our hypotheses. At Step 1, T2 behavior was regressed onto T1 overall attitude. Step 2a added cognitive-affective inconsistency and the overall Attitude × Cognitive-Affective Inconsistency interaction to overall attitude from Step 1. Step 2b added cognitive-affective ambivalence and the overall Attitude × Cognitive-Affective Ambivalence interaction to evaluative attitude from Step 1. Step 3 added cognitive-affective ambivalence and the overall Attitude × Cognitive-Affective Ambivalence interaction to variables from Step 2a. Step 2a tests the moderating effects of cognitive-affective inconsistency, Step 2b tests the moderating effects of cognitive-affective ambivalence, and Step 3 tests both moderating effects simultaneously. Mean-centered scores were used in the regression to minimize problems of multicollinearity (Aiken & West, 1991). Simple slopes analyses (Aiken & West, 1991) were used to explore any significant interaction terms by examining the unstandardized regression slope for T1 overall attitude at low (*M* − 1*SD*), mean, and high (*M* + 1*SD*) levels of the moderator.

### Results

Table 1 shows that the measures of behavior, overall attitude, cognitive-affective inconsistency, and cognitive-affective ambivalence showed reasonable variation for both eating a low-fat diet (above diagonal) and eating five portions of fruit and vegetables per day (below diagonal). Overall attitudes were relatively positive for each behavior, although cognitive-affective inconsistency and ambivalence were relatively low (Table 1). Overall attitude was a significant positive predictor for both behaviors. Overall attitude was significantly negatively related to cognitive-affective inconsistency for both behaviors. Cognitive-affective inconsistency and ambivalence were strongly significantly positively intercorrelated for both behaviors.

**Table 1.** Descriptive Data and Intercorrelation of Measures in Study 1 (Eating a Low-Fat Diet Above Diagonal, *N* = 136; Eating Five Portions of Fruit and Vegetables Below Diagonal, *N* = 135).

Measures	B	OA	CAI	CAA	*M*	*SD*
T2 behavior (B)	1.000	0.261**	−0.124	−0.093	37.228	8.119
Overall attitude (OA)	0.228***	1.000	−0.246**	−0.257**	5.380	1.285
Cognitive-affective inconsistency (CAI)	−0.224**	−0.325***	1.000	0.668***	1.688	1.287
Cognitive-affective ambivalence (CAA)	−0.193*	−0.154	0.774***	1.000	−1.384	1.190
*M*	4.070	5.575	1.296	−1.543		
*SD*	2.102	1.562	1.432	1.170		

*Note*. M = mean; SD = standard deviation.

* p < .05. **p < .01. ***p < .001.

Table 2 shows the results of the moderated regression analyses. In relation to eating a low-fat diet (Table 2, left-hand panel), at Step 1 overall attitude was a significant positive predictor of behavior. At Step 2a, overall attitude was a significant positive predictor, cognitive-affective inconsistency was a non-significant negative predictor, and the interaction between overall attitude and inconsistency was a significant negative predictor, *B* = −1.129, 95% CI = [−1.901, −0.356]. Simple slopes analyses of the overall Attitude × Cognitive-Affective Inconsistency interaction at Step 2a (Table 2), indicated that at low levels of inconsistency, overall attitude was strongly related to behavior, *B* = 2.943, *SE* = .719, *p* < .001, while at moderate levels of inconsistency overall attitude was less strongly related to behavior, *B* = 1.490, *SE* = .531, *p* = .006, and at high levels of inconsistency overall attitude was unrelated to behavior, *B* = 0.037, *SE* = .749, *p* = .964. At Step 2b, the interaction between overall attitude and cognitive-affective ambivalence was not significant and did not explain a significant additional increment of variance in behavior. At Step 3, the addition of cognitive-affective ambivalence and the interaction between overall attitude and cognitive-affective ambivalence (to Step 2a) did not explain significant additional variance in behavior and the added interaction was not significant. Overall attitude remained a significant positive predictor and the interaction between overall attitude and cognitive-affective inconsistency remained as a significant negative predictor at Step 3.

**Table 2.** Moderated Linear Regression of Behavior Onto Overall Attitude, Cognitive-Affective Inconsistency, Cognitive-Affective Ambivalence, and Interactions in Study 1.

	Low-fat diet			Five portions of fruit and vegetables		
Predictors	*B*	*SE B*	β	*B*	*SE B*	β
1. Overall attitude (OA)	1.650	.527	.261**	0.306	.114	.228**
2a. OA	1.490	.531	.236**	0.300	.120	.223*
Cognitive-affective inconsistency (CAI)	−0.409	.530	−.065	−0.272	.128	−.185*
OA × CAI	−1.129	.394	−.233**	−0.185	.075	−.210*
2b. OA	1.524	.554	.241**	0.264	.115	.197*
Cognitive-affective ambivalence (CAA)	−0.369	.623	−.054	−0.329	.161	−.183*
OA × CAA	−0.455	.494	−.082	−0.074	.100	−.066
3. OA	1.576	.542	.249**	0.379	.127	.281**
CAI	−0.529	.707	−.084	−0.147	.202	−.100
OA × CAI	−1.480	.501	−.306**	−0.301	.105	−0.340**
CAA	0.312	.814	.046	−0.130	.246	−0.072
OA × CAA	0.706	.617	.126	0.202	.136	.178

*Note.* Eating a low-fat diet, *N* = 136: Step 1, Δ*F*(1, 134) = 9.81, *p* = .002, Δ*R*^2^ = .068; Step 2a, Δ*F*(2, 132) = 4.40, *p* = .014, Δ*R*^2^ = .058; Step 2b, Δ*F*(2, 132) = 0.47, *p* = .624, Δ*R*^2^ = .007; and Step 3, Δ*F*(2, 130) = 0.66, *p* = .521, Δ*R*^2^ = .009. Eating five portions of fruit and vegetables, *N* = 135: Step 1, Δ*F*(1, 133) = 7.27, *p* = .008, Δ*R*^2^ = .052; Step 2a, Δ*F*(2, 131) = 4.88, *p* = .009, Δ*R*^2^ = .066; Step 2b, Δ*F*(2, 131) = 2.10, *p* = .127, Δ*R*^2^ = .029; and Step 3, Δ*F*(2, 129) = 1.58, *p* = .210, Δ*R*^2^ = .021.

* *p* < .05. **p < .01. ***p < .001.

In relation to eating five fruits and vegetables per day (Table 2, right-hand panel), at Step 1 overall attitude was a significant positive predictor of behavior. At Step 2a, overall attitude was a significant positive predictor, cognitive-affective inconsistency was a significant negative predictor of behavior, and the interaction between overall attitude and inconsistency was a significant negative predictor, *B* = −0.021, 95% CI = [−0.037, −0.005]. Simple slopes analyses of the Overall Attitude × Cognitive-Affective Inconsistency interaction at Step 2a (Table 2), indicated that at low levels of inconsistency, overall attitude was strongly related to behavior, *B* = 0.063, *SE* = .020, *p* = .002, while at moderate levels of inconsistency overall attitude was less strongly related to behavior, *B* = 0.033, *SE* = .013, *p* = .014, and at high levels of inconsistency overall attitude was unrelated to behavior, *B* = 0.004, *SE* = .016, *p* = .809. At Step 2b, although overall attitude was a significant positive predictor and cognitive-affective ambivalence was a significant negative predictor, the interaction between overall attitude and cognitive-affective ambivalence was not significant and this step did not explain a significant additional increment of variance in behavior. At Step 3, the addition of cognitive-affective ambivalence and the interaction between overall attitude and cognitive-affective ambivalence did not explain significant additional variance in behavior compared with Step 2a and the added interaction was not significant. Overall attitude remained a significant positive predictor and the interaction between overall attitude and cognitive-affective inconsistency remained as a significant negative predictor in Step 3.

### Discussion

The results from Study 1 were in line with predictions. For both eating a low-fat diet and eating five portions of fruit and vegetables per day, cognitive-affective inconsistency significantly moderated the overall attitude–behavior relationship (Table 2). In particular, lower compared with higher levels of inconsistency were associated with significantly stronger impacts of overall attitude on behavior. Cognitive-affective ambivalence did not moderate the overall attitude–behavior relationship and controlling for it did not remove the moderating effects for cognitive-affective inconsistency.

Although Study 1 provides support for our key predictions, there are a number of potential weaknesses. First, we were not able to control for past behavior as this was not measured. Second, the behavioral measure was self-report and so open to various self-report biases. Third, the time interval between T1 and T2 was relatively modest (1 month) thus precluding any test of whether our moderating effects of cognitive-affective inconsistency persist over more prolonged intervals. Study 2 was intended to address these potential weaknesses and assess the generalizability of our findings to a different behavior, sample, and overall attitude to behavior time delay.

## Study 2

Study 2 focused on smoking initiation in adolescents. The current data have not been previously reported but are part of a larger randomized controlled trial testing implementation intentions as an intervention to reduce smoking initiation (Conner et al., 2013, 2019) The current analyses control for intervention condition which did not moderate any of the relationships reported. A number of measures not reported here were also included (e.g., intentions; normative beliefs; perceived behavioral control). The full T1 questionnaire is provided as an online appendix. The data are available at https://osf.io/unz4s/?view_only=591a16ab02f04a49bc7947636ca6775b. The University of Leeds, UK (Faculty of Medicine), ethical review committee approved the study.

### Method

#### Sample

Adolescents from a single school year in 45 schools in the United Kingdom participated in the study and completed questionnaires each year. Responses were completed anonymously and matched across time points (T1–T2) using a personally generated code. The data reported here focus on questionnaire measures when participants were aged 14–15 years (T1) and an objective smoking measure was taken 12 months later when participants were 15–16 years of age (T2). There were 6,387 participants who completed all measures at T1. Based on a personally generated code it was possible to match 4,933 participants across T1 and T2. Compared with the unmatched sub-sample, the matched sub-sample had a less positive overall attitude toward smoking, *F*(1, 6,385) = 77.45, *p* < .001; higher cognitive-affective inconsistency, *F*(1, 6,385) = 30.05, *p* < .001; lower cognitive-affective ambivalence, *F*(1, 6,385) = 7.04, *p* = .008; and were less likely to be smokers at T1, *F*(1, 6,385) = 22.17, *p* < .001. There were 2,378 boys and 2,554 girls in the final sample, age: *M* = 14.19 years, *SD* = 0.39.

#### Measures

Overall attitude toward smoking was assessed at T1 by a single semantic differential item (“For me smoking would be . . .,” “*bad-good*”). Cognitive evaluation was assessed at T1 by three semantic differential items (“For me smoking would be . . .,” “*harmful-beneficial,”* “*foolish-wise*,” “*unhealthy-healthy*”; α = .62). Affective evaluation was assessed at T1 by three semantic differential items (“For me smoking would be . . .,” “*unpleasant-pleasant*,” “*unenjoyable-enjoyable*,” “*not fun-fun*”; α = .93). Each item was scored 1 to 5 (higher scores indicated more positive judgments).

Cognitive-affective inconsistency was computed as the absolute difference between cognitive and affective evaluations (range 0–4). A measure of cognitive-affective ambivalence was computed in the same way as in Study 1 using the bipolar measures of cognitive and affective evaluations combined using the Griffin equation. Patterns of responses on cognitive and affective evaluations that were not oppositely valenced were coded as non-ambivalent (coded −1 to reflect the lowest level of ambivalence possible based on the positive score at the most extreme [2] and the negative score at the mid-point [0] or vice versa in the Griffin equation). The cognitive-affective ambivalence scores ranged between −1 and +2.

Past behavior was assessed at T1 using a self-report measure (adolescents ticked one of “I have never smoked”; “I have only tried smoking once”; “I used to smoke sometimes, but I never smoke cigarettes now; “I sometimes smoke cigarettes now, but I don’t smoke as many as one a week”; “I usually smoke between one and six cigarettes a week”; “I usually smoke more than six cigarettes a week”). This was coded 0 for the first response and 1 for all other responses. T2 behavior was assessed using a measure of breath carbon monoxide (CO) levels (using Micro+ Smokerlyzer^®^ CO Monitor, Bedfont Scientific Limited, Kent, England). Such measures are reliable and valid ways of distinguishing regular cigarette smoking from never or occasional smoking (Jarvis et al., 1987). Scores were highly skewed toward not smoking and therefore dichotomized at the median (0 = “0–1 ppm of CO coded as no smoking”; 1 = “>1 ppm of CO coded as smoking”; see Conner & Higgins, 2010 for a similar approach).

#### Analysis

The distribution of the measures and their intercorrelations was first examined. Moderated logistic regression analysis was then used to test the predictions. At Step 1, T2 behavior was regressed onto T1 overall attitude. Steps 2a, 2b, and 3 were the same as in Study 1. Step 4 added past behavior to variables from Step 3. We used mean-centered scores to minimize multicollinearity and used simple slopes analyses to explore significant interactions (Aiken & West, 1991).

### Results

Table 3 (above diagonal) shows that the variables had reasonable variance but that overall attitude was skewed toward a negative attitude, although this should not affect the logistic regressions used in subsequent analyses. Cognitive-affective inconsistency and ambivalence were generally low. T2 behavior showed a small but significant positive correlation with overall attitude and past behavior and a small but significant negative correlation with inconsistency. Overall attitude showed medium sized significant correlations with cognitive-affective inconsistency (negative) and ambivalence (positive) and also past behavior (positive). Cognitive-affective inconsistency had a small but significant positive correlation with cognitive-affective ambivalence (Table 3, above diagonal).

**Table 3.** Descriptive Data and Intercorrelation of Measures (Study 2 on Smoking Initiation, *N* = 4,933 Above Diagonal; Study 3 on Physical Activity, *N* = 909 Below Diagonal).

Measures	B	OA	CAI	CAA	PB	*M*	*SD*
T2 behavior (B)	1.000	0.066***	−0.049***	0.019	0.068***	0.631	0.483
Overall attitude (OA)	0.366***	1.000	−0.287***	0.216***	0.342***	1.188	0.612
Cognitive-affective inconsistency (CAI)	−0.295***	−0.140***	1.000	0.070***	−0.250***	1.112	0.467
Cognitive-affective ambivalence (CAA)	−0.279***	−0.354***	0.745***	1.000	0.182***	−0.967	0.204
T1 past behavior	0.654***	0.395***	−0.400***	−0.358***	1.000	0.243	0.429
*M*	0.052	2.924	2.554	−2.067	0.015		
*SD*	0.761	2.200	2.281	1.818	0.753		

*Note*. M = mean; SD = standard deviation.

* *p* < .05. **p < .01. ***p < .001.

Table 4 shows the results of the moderated logistic regression analyses for predicting behavior. At Step 1, a significant increment in the variance in behavior was explained and overall attitude was a significant positive predictor of behavior. At Step 2a, a further significant increment in the variance in behavior was explained and overall attitude and the interaction between overall attitude and cognitive-affective inconsistency, OR = 0.818, 95% CI = [0.692, 0.966], were significant predictors of behavior. To explore the nature of the interaction, we examined the logistic slopes for overall attitude at low, moderate, and high levels of cognitive-affective inconsistency. This indicated that overall attitude was a stronger predictor of behavior at low, *B* = 0.252, *SE* = .059, *p* < .001, OR = 1.286, 95% CI = [1.147, 1.444], compared with moderate, *B* = 0.154, *SE* = .059, *p* = .009, OR = 1.167, 95% CI = [1.039, 1.310], inconsistency, while at high levels of inconsistency overall attitude was not significantly related to behavior, *B* = 0.057, *SE* = .082, *p* = .486, OR = 1.058, 95% CI = [0.902, 1.242].

**Table 4.** Moderated Logistic Regression of Behavior Onto Overall Attitude, Cognitive-Affective Inconsistency, Cognitive-Affective Ambivalence, Interactions, and Past Behavior in Study 2 (*N* = 4,933).

Step/predictor	*B*	*SE B*	Odds ratio	95% CI
1. Overall attitude (OA)	0.240	.054	1.271***	[1.143, 1.414]
2a. OA	0.152	.060	1.164*	[1.035, 1.308]
Cognitive-affective inconsistency (CAI)	−0.113	.068	0.893	[0.782, 1.021]
OA × CAI	−0.201	.085	0.818*	[0.692, 0.966]
2b. OA	0.257	.058	1.293***	[1.155, 1.448]
Cognitive-affective ambivalence (CAA)	0.136	.189	1.146	[0.790, 1.660]
OA × CAA	−0.203	.144	0.816	[0.615, 1.082]
3. OA	0.145	.068	1.156*	[1.011, 1.321]
CAI	−0.117	.069	0.889	[0.777, 1.018]
OA × CAI	−0.204	.094	0.816*	[0.679, 0.981]
CAA	0.142	.189	1.153	[0.796, 1.670]
OA × CAA	−0.043	.158	0.958	[0.703, 1.306]
4. OA	0.094	.070	1.098	[0.958, 1.260]
CAI	−0.082	.070	0.921	[0.804, 1.057]
OA × CAI	−0.214	.093	0.807*	[0.673, 0.969]
CAA	0.050	.191	1.051	[0.722, 1.530]
OA × CAA	0.003	.158	1.003	[0.735, 1.368]
Past behavior (PB)	0.222	.078	1.248**	[1.072, 1.454]

*Note.* Step 1: Δχ^2^(1) = 21.29, *p* < .001, ΔNagelkerke *R*^2^ = .006; Step 2a: Δχ^2^(2) = 9.89, *p* = .007, ΔNagelkerke *R*^2^ = .002; Step 2b: Δχ^2^(2) = 1.94, *p* = .379, ΔNagelkerke *R*^2^ = .000; Step 3: Δχ^2^(2) = 0.60, *p* = .742, ΔNagelkerke *R*^2^ = .001; Step 4: Δχ^2^(1) = 8.20, *p* = .004, ΔNagelkerke *R*^2^ = .002. SE = standard error.

* *p* < .05. **p < .01. ***p < .001.

At Step 2b, the interaction between overall attitude and cognitive-affective ambivalence was not significant and no significant additional increment of variance in behavior was explained. At Step 3, the addition of cognitive-affective ambivalence and the interaction between overall attitude and cognitive-affective ambivalence to the variables in Step 2a did not explain a significant increment in the variance in behavior. Only overall attitude and the interaction between overall attitude and cognitive-affective inconsistency remained significant at this step. At Step 4, the addition of past behavior explained a further significant increment in the variance in behavior. Only past behavior and the interaction between overall attitude and cognitive-affective inconsistency were significant at this step.

Further analysis examined whether the interaction between overall attitude and cognitive-affective inconsistency remained significant when controlling for past behavior but not cognitive-affective ambivalence or the interaction between overall attitude and cognitive-affective ambivalence (i.e., Step 2a in Table 4 with past behavior added). In this analysis, the interaction between overall attitude and cognitive-affective inconsistency did remain significant, *B* = −0.208, *SE* = .085, *p* = .014, OR = 0.812, 95% CI = [0.688, 0.959].

### Discussion

The results from Study 2 were mostly consistent with Study 1. Cognitive-affective incosnsistency significantly moderated the overall attitude–behavior relationship. In particular, lower compared with higher levels of inconsistency were associated with overall attitude being a stronger predictor of behavior. This pattern remained and was little changed when also controlling for cognitive-affective ambivalence (Step 3) and past behavior (Step 4, Table 4). Importantly, Study 2 used an objective measure of behavior. Cognitive-affective ambivalence did not moderate the overall attitude–behavior relationship.

Although Study 2 provided further support for our predictions, there are a number of potential weaknesses. Most importantly, our measure of overall attitude was based on a single item potentially reducing reliability (as was the measure of cognitive evaluation in Study 1). In addition, our measures of cognitive and affective evaluations were based on a limited number of items (i.e., three items). Study 3 was designed to address these weaknesses and provide a further test of our predictions in a different behavior, sample, and attitude to behavior time delay.

## Study 3: Physical Activity

Study 3 focused on physical activity. A number of measures not reported here were also included (e.g., intentions, anticipatory affect, goal priority, wants, and shoulds). The full questionnaires (T1 and T2) are provided as an online appendix. The data are available here: https://osf.io/unz4s/?view_only=591a16ab02f04a49bc7947636ca6775b. The University of Leeds, UK, ethical review committee approved the study.

### Method

#### Sample

Participants were recruited via Prolific, an online database of participants interested in taking part in research from a range of academic areas. From a database of approximately 26,000 potential participants, a total of 1,007 individuals were recruited and completed the T1 questionnaire. A total of 909 participants (90% response rate) subsequently completed the four-week follow-up questionnaire (T2) and could be matched to T1 data. Data were collected between May and June 2018. Participants were paid £4.30 (US$6.03 at time of recruitment) for completing both questionnaires. There were 361 males and 540 females (eight missing), mean age 34.9 years (*SD* = 10.81). The 909 analyzed respondents did not differ from the 98 excluded on baseline measures of overall attitude, cognitive-affective inconsistency, or past behavior, *p* > .43, but reported less cognitive-affective ambivalence, *F*(1, 1005) = 4.48, *p* = .035.

#### Measures

Overall attitude was assessed at T1 by eight evaluative split semantic differential items (bad–good, negative–positive, unfavorable–favorable, and unimportant–important; Thompson et al., 1995) tapping positive (e.g., “My engaging in the recommended levels of physical activity each week over the next month would be, *not at all positive-extremely positive*,” scored 1–7, α = .86) and negative (e.g., “My engaging in the recommended levels of physical activity each week over the next month would be, *not at all negative-extremely negative*,” scored 1–7, α = .85) evaluations. The positive and negative evaluations were each averaged and a difference score computed (i.e., mean positive evaluation—mean negative evaluation) such that overall attitude scores ranged from −6 to +6.

Cognitive and affective evaluations were assessed in a similar way based on split semantic differential measures. Cognitive evaluation was assessed at T1 by eight split semantic differential items tapping positive (useful, beneficial, healthy, and valuable; scored 1–7, α = .91) and negative (useless, harmful, unhealthy, and worthless; scored 1–7, α = .88) reactions. The positive and negative reactions were averaged and a difference score computed (i.e., mean positive evaluation—mean negative evaluation) such that cognitive evaluation scores ranged from −6 to +6. Affective evaluation was assessed at T1 by eight split semantic differential items tapping positive (enjoyable, pleasurable, exciting, and agreeable; scored 1–7, α = .91) and negative (unenjoyable, unpleasurable, boring, and disagreeable; scored 1–7, α = .90) reactions. The positive and negative reactions were averaged and a difference score computed (i.e., mean positive evaluation—mean negative evaluation) such that affective evaluation scores ranged from −6 to +6.

Cognitive-affective inconsistency was computed as the absolute difference between cognitive and affective evaluations (scored between 0 and 12).

Cognitive-affective ambivalence was computed in the same manner as used in Studies 1 and 2 using the created bipolar measures of cognitive and affective evaluations combined using the Griffin equation. Patterns of responses on cognitive and affective evaluations that were not of opposing valence were coded as non-ambivalent (coded −3 to reflect the lowest level of ambivalence possible based on the positive score at the most extreme [6] and the negative score at the mid-point [0], or vice versa in the Griffin equation). The cognitive-affective ambivalence scores ranged between −3 and +6.

Behavior was assessed using six items at both time points. Items were a mixture of closed ended (e.g., “How frequently did you engage in the recommended levels of physical activity each week over the last month?, *never—always*,” scored 1–7) and open-ended (e.g., “Over the past month, how many weeks did you engage in the recommended levels of physical activity?, *____ weeks*”) questions. These were converted to *z*-scores and averaged to create a measure of past behavior (based on items from the T1 questionnaire, α = .80) and future behavior (based on items from the T2 questionnaire, α = .81).

#### Analysis

The analysis followed the same procedures as used in Study 2.

### Results

Table 3 (below diagonal) shows that the measures of behavior, overall attitude, cognitive-affective inconsistency, cognitive-affective ambivalence, and past behavior had reasonable variance. Overall attitude was slightly positive and cognitive-affective inconsistency and ambivalence were low. Overall attitude and past behavior were significant positive predictors of behavior, while cognitive-affective inconsistency and ambivalence were significant negative predictors. Overall attitude had a moderate significant positive correlation with past behavior, a small significant negative correlation with cognitive-affective inconsistency, and a moderate negative correlation with cognitive-affective ambivalence. Cognitive-affective inconsistency and ambivalence had a strong significant positive intercorrelation.

Table 5 shows the results of the moderated regression analyses to predict behavior at T2. At Step 1, overall attitude was a significant positive predictor of behavior. At Step 2a, overall attitude was a significant positive predictor, while cognitive-affective inconsistency and the interaction between overall attitude and cognitive-affective inconsistency, *B* = −0.017, 95% CI = [−0.026, −0.008], were significant negative predictors. Simple slopes analyses of the overall Attitude × Cognitive-Affective Inconsistency interaction at Step 2a (Table 5) indicated that at low levels of inconsistency, overall attitude was strongly related to behavior, *B* = 0.149, *SE* = .014, *p* < .001; while at moderate levels of inconsistency, overall attitude was less strongly related to behavior, *B* = 0.110, *SE* = .010, *p* < .001; and at high levels of inconsistency, then overall attitude was even less strongly related to behavior, *B* = 0.071, *SE* = .015, *p* < .001.

**Table 5.** Moderated Linear Regression of Behavior Onto Overall Attitude, Cognitive-Affective Inconsistency, Cognitive-Affective Ambivalence, Interactions, and Past Behavior in Study 3 on Physical Activity (*N* = 909).

Step/predictor	*B*	*SE B*	β
1. Overall attitude (OA)	0.127	.011	.366***
2a. OA	0.110	.010	.317***
Cognitive-affective inconsistency (CAI)	−0.091	.010	−.272***
OA × CAI	−0.017	.004	−.117***
2b. OA	0.097	.011	.281***
Cognitive-affective ambivalence (CAA)	−0.106	.017	−.252***
OA × CAA	−0.026	.007	−.133***
3. OA	0.109	.012	.316***
CAI	−0.090	.015	−.270***
OA × CAI	−0.017	.006	−.114**
CAA	−0.002	.024	−.005
OA × CAA	−0.001	.010	−.005
4. OA	0.043	.010	.125***
CAI	−0.017	.013	−.052
OA × CAI	−0.007	.005	−.047
CAA	0.000	.020	−.001
OA × CAA	0.001	.008	.004
Past behavior (PB)	0.586	.030	.579***

*Note.* Step 1: Δ*F*(1, 907) = 140.59, *p* < .001, Δ*R*^2^ = .134; Step 2a: Δ*F*(2, 905) = 41.92, *p* < .001, Δ*R*^2^ = .073; Step 2b: Δ*F*(2, 905) = 20.345, *p* < .001, Δ*R*^2^ = .037; Step 3: Δ*F*(2, 903) = 0.01, *p* = .994, Δ*R*^2^ = .000; and Step 4: Δ*F*(1, 902) = 385.94, *p* < .001, Δ*R*^2^ = .237.

* *p* < .05. **p < .01. ***p < .001.

At Step 2b, overall attitude was a significant positive predictor, while cognitive-affective ambivalence and the interaction between overall attitude and cognitive-affective ambivalence, *B* = −0.026, 95% CI = [−0.040, −0.012], were significant negative predictors. Simple slopes analyses of the overall Attitude × Cognitive-Affective Ambivalence interaction at Step 2b (Table 5) indicated that at low levels of ambivalence, overall attitude was strongly related to behavior, *B* = 0.149, *SE* = .014, *p* < .001; while at moderate levels of ambivalence, overall attitude was less strongly related to behavior, *B* = 0.097, *SE* = .011, *p* < .001; and at high levels of ambivalence, then overall attitude was even less strongly related to behavior, *B* = 0.071, *SE* = .015, *p* < .001.

At Step 3, overall attitude remained as a significant positive predictor, cognitive-affective inconsistency and the interaction between overall attitude and cognitive-affective inconsistency remained as significant negative predictors, while cognitive-affective ambivalence and the interaction between overall attitude and cognitive-affective ambivalence were non-significant predictors. At Step 4, overall attitude and past behavior were the only significant predictors.

Further analyses examined whether the interaction between overall attitude and each of the two moderators (cognitive-affective inconsistency or ambivalence) considered separately remained significant when controlling for past behavior but not the other moderator (i.e., Step 2a or Step 2b in Table 5 each with past behavior added). In these analyses, the interaction between overall attitude and cognitive-affective inconsistency was marginally significant, *B* = −0.006, *SE* = .004, β = −.044, *p* = .088, while the interaction between overall attitude and cognitive-affective ambivalence was not significant, *B* = −0.007, *SE* = .006, β = −.038, *p* = .219.

### Discussion

The results from Study 3 were generally consistent with both Studies 1 and 2. Cognitive-affective inconsistency significantly moderated the overall attitude–behavior relationship (Step 2a, Table 5). In particular, lower compared with higher levels of inconsistency were associated with stronger impacts of overall attitude on behavior. This pattern remained when controlling for cognitive-affective ambivalence and the interaction between overall attitude and ambivalence (Step 3, Table 5) but not when also controlling for past behavior (Step 4, Table 5). Cognitive-affective ambivalence also significantly moderated the overall attitude–behavior relationship (Step 2b, Table 5), although this effect was attenuated and became non-significant when cognitive-affective inconsistency and its interaction with overall attitudes were included in the equation (Step 3, Table 5). This supports the idea that cognitive-affective inconsistency explains the moderating effect of cognitive-affective ambivalence on the overall attitude–behavior relationship.

## Meta-Analysis of Studies 1 to 3

In a final analysis we used meta-analysis (on frequency-weighted effect sizes with fixed effects in JASP; https://jasp-stats.org/) to help understand the overall effects across studies. This indicated the correlation between cognitive-affective inconsistency and ambivalence across studies to be of a large magnitude, *r_+_* = .563, *SE* = .145, 95% CI = [0.279, 0.847], *p* < .001. The significant moderating effect of cognitive-affective inconsistency on the overall attitude to behavior relationship was similar across the four tests in the three studies. In each case we observed a significant negative interaction between cognitive-affective inconsistency and overall attitude (after controlling for main effects) in predicting behavior (Step 2a in Tables 2, 4, and 5). Simple slopes analyses indicated that as cognitive-affective inconsistency increased the impact of overall attitude on behavior decreased. Perhaps not surprisingly, meta-analysis indicated an overall significant effect for the cognitive-affective inconsistency by overall attitude interaction across studies (individual test), *B* = −0.018, *SE* = .004, 95% CI = [−0.025, −0.011], *p* < .001.

The effects in each study were also similar in terms of whether the cognitive-affective inconsistency by overall attitude interaction remained significant after controlling for cognitive-affective ambivalence and its interaction with overall attitude (Step 3 in Tables 2, 4, and 5). Meta-analysis indicated that the overall effect size for the interaction was significant when studies were combined (simultaneous test), *B* = −0.067, *SE* = .026, 95% CI = [−0.119, −0.015], *p* = .011. This supports the idea that cognitive-affective inconsistency moderates the overall attitude–behavior relationship even when controlling for the related measure of cognitive-affective ambivalence.

In relation to cognitive-affective ambivalence, we only observed a significant negative interaction between cognitive-affective ambivalence and overall attitude (after controlling for the main effects) in predicting behavior in Study 3 (Step 2b in Tables 2, 4, and 5). Simple slopes analyses in Study 3 indicated that as cognitive-affective ambivalence increased the impact of overall attitude on behavior decreased. However, meta-analysis indicated that there was a significant average negative effect for the cognitive-affective ambivalence by overall attitude interaction across studies (individual test), *B* = −0.021, *SE* = .006, 95% CI = [−0.033, 0.001], *p* < .001. The studies were similar in showing the cognitive-affective ambivalence by overall attitude interaction was not significant after controlling for cognitive-affective inconsistency and its interaction with overall attitude (Step 3 in Tables 2, 4, and 5). Meta-analysis also indicated that the overall effect size for the interaction was not significant when studies were combined (simultaneous test), *B* = −0.006, *SE* = .008, 95% CI = [−0.010, 0.023], *p* = .465. This supports the idea that although cognitive-affective ambivalence moderates the overall attitude to behavior relationship this effect becomes non-significant when controlling for the related measure of cognitive-affective inconsistency.

## General Discussion

This research focused on cognitive-affective inconsistency and ambivalence as potential moderators of the overall attitude–behavior relationship. Across three prospective studies in four different behaviors and three different populations with differing time delays a fairly comparable set of findings emerged. In each study the correlation between cognitive-affective inconsistency and ambivalence was significant and positive but ranged from weak to strong in magnitude, *r* = .070 to .774 (Tables 1 and 3). Meta-analysis indicated the overall correlation between the two was significant, positive and of large magnitude across studies, *r_+_* = .563, 95% CI = [0.279, 0.847]. In each study, cognitive-affective inconsistency significantly moderated the relationship between overall attitude and behavior (individual tests: Step 2a, Tables 2, 4, and 5). In each case, increasing cognitive-affective inconsistency was associated with a lower overall attitude–behavior relationship. The meta-analysis indicated that the effect across studies was significant, *B* = −0.018, 95% CI = [−0.025, −0.011]. These findings are consistent with cognitive-affective inconsistency being regarded as a measure of attitude strength (Krosnick & Petty, 1995). Only in Study 3 did cognitive-affective ambivalence significantly moderate the relationship between overall attitude and behavior (individual tests: Step 2b, Tables 2, 4, and 5). In this case, increasing cognitive-affective ambivalence was associated with a lower overall attitude–behavior relationship. However, the meta-analysis did indicate that the effect across studies was significant, *B* = −0.021, 95% CI = [−0.033, −0.001]. The meta-analysis findings are consistent with cognitive-affective ambivalence also being regarded as a measure of attitude strength (Krosnick & Petty, 1995).

It is notable that the effect sizes in the meta-analyses for cognitive-affective inconsistency and ambivalence as moderators are of very similar magnitude. However, the key test of the relative effects of cognitive-affective inconsistency and ambivalence is their power to moderate overall attitude–behavior relationships when controlling for one another (simultaneous tests). In each study, when considered simultaneously, cognitive-affective inconsistency significantly moderated the relationship between overall attitude and behavior while cognitive-affective ambivalence did not (Step 3, Table 2, 4, and 5). The meta-analysis including both moderators showed similar results. Cognitive-affective inconsistency was a significant moderator, *B* = −0.021, 95% CI = [−0.033, −0.011], while cognitive-affective ambivalence was not, *B* = −0.006, 95% CI = [−0.010, 0.023]. These findings support the idea that cognitive-affective inconsistency is the more important moderator of the overall attitude–behavior relationship, that is, that inconsistency explains the effects of ambivalence on the overall attitude–behavior relationship.

We would argue that a key contribution of the present research is in showing that inconsistency between cognitive and affective evaluations matters (whether or not these judgments are of opposing valence or not) in relation to the strength of the overall attitude. For example, cognitive-affective ambivalence focuses on the more limited situation where cognitive evaluations are positive and affective evaluations are negative (or vice versa). In contrast, cognitive-affective inconsistency also covers the broader situation where cognitive and affective evaluations are similarly valenced but differ in extremity (Figure 1). The fact that we find cognitive-affective inconsistency to play a more important role within our regression models than cognitive-affective ambivalence suggests that it is important to think about cognitive-affective inconsistency as a measure of attitude strength in a broader way, not limited to situations where the components are in evaluative opposition (i.e., opposing valences). In fact, one could even argue that higher levels of cognitive-affective inconsistency *without* holding evaluatively opposing thoughts and feelings (not captured by cognitive-affective ambivalence) is quite common in daily life. Think of falling in love for example, where the affective component sometimes is not so much in *opposition* with the cognitive component, it is just that the reasons we can give for loving this person often do not do justice to the feeling we have when we see them. In fact, on the basis of their definitions it seems plausible to assume that cognitive-affective inconsistency occurs more often than cognitive-affective ambivalence further adding to the importance of the present findings.

Cognitive consistency theories (Festinger, 1957; Heider, 1958) and the tripartite model of attitude structure (Breckler, 1994; Rosenberg & Hovland, 1960) would suggest that cognitive and affective evaluations of the same attitude object should generally be similarly valenced and at least moderately positively correlated. In addition, the fact that each component may be based on (or derived from) the other (Lavine et al., 1998; Zajonc & Markus, 1982) likely enhances the relationship between the two. This might suggest that when cognitive and affective evaluations are inconsistent, despite these various processes prompting consistency, this may be important and consequential for the strength of an overall attitude derived from them.

The current research does not provide definitive insights into why cognitive-affective inconsistency is more important than cognitive-affective ambivalence in moderating the overall attitude–behavior relationship. We noted in the introduction that inconsistency taps situations where cognitive and affective evaluations differ from one-another whether or not they have opposing valences. This might suggest that any inconsistency between cognitive and affective evaluation is the most important characteristic rather than being of opposing valence in relation to the strength of the resulting overall attitude. However, it would be useful to see the current findings replicated for a broader range of behaviors and in both correlational and experimental studies before too much reliance is placed on them.

Future research might also usefully explore awareness of cognitive-affective inconsistency and potential consequences for re-evaluation of overall attitude. We did not assess felt cognitive-affective inconsistency (e.g., “I have mixed thoughts and feelings about behavior x”) or ambivalence (e.g., “I have positive [negative] thoughts but negative [positive] feelings about behavior x”; see Conner & Sparks, 2002). Previous research has shown such measures of felt ambivalence to be less consistent moderators of attitude–behavior relations compared with measures of potential ambivalence (Conner & Armitage, 2008) perhaps because the felt ambivalence prompts re-evaluation of attitudes and behavior (van Harreveld et al., 2009, 2015). Future research might usefully assess whether awareness of cognitive-affective inconsistency does indeed prompt re-evaluation of attitudes and whether this results in overall attitudes that are more aligned with cognitive or affective evaluations (the work of Lavine et al., 1998 might suggest the latter). Finally, an interesting further possibility for future research would be to explore the effects of cognitive-affective inconsistency where that inconsistency does not overlap with cognitive-affective ambivalence (i.e., cognitive and affective evaluations have the same valence) versus where it does overlap with cognitive-affective ambivalence (i.e., cognitive and affective evaluations do not have the same valence). As we noted in the introduction, testing the moderating effects of cognitive-affective inconsistency and ambivalence simultaneously suggests that it is not simply the case that it is the cognitive and affective evaluations being of opposing valence that drives the moderation of the overall attitude–behavior relationship.

The present research has a number of strengths including replication of effects across four behaviors in three different (and large) samples, differing time intervals between overall attitude and behavior measurement, controlling for past behavior (Studies 2 and 3), and using an objective measure of behavior (Study 2). There are also a number of weaknesses. First, Studies 1 and 2 both employed single-item measures of some constructs. Second, cognitive-affective ambivalence was coded as low when the cognitive and affective evaluations did not have opposing valences regardless of extremity and differences of the two evaluations. This might be justified based on ambivalence being defined in relation to opposing valences and reduces the overlap between ambivalence and inconsistency. Nevertheless it is worth noting that this diverges from approaches such as the Gradual Threshold Model (Priester & Petty, 1996) that imply that, even when there are only positive or only negative evaluations, ambivalence reduces to the degree that there are many rather than fewer valenced evaluations. Third, the present data do not provide definitive tests of the potential mechanisms underlying the moderating effects of cognitive-affective inconsistency on the overall attitude–behavior relationship. In the introduction we specifically referred to effects of inconsistency on the stability of overall attitude as one mechanism. Fabrigar et al. (2005) refer to this as a prediction mechanism and distinguish it from an influence mechanism (i.e., inconsistency being a marker for how good a guide to behavior the overall attitude should be) of how attitude structure might influence the attitude–behavior relationship. Research that tests whether it is one or both these mechanisms (or indeed other mechanisms) that operate in relation to the moderating effects of cognitive-affective inconsistency on overall attitude–behavior relationships would be useful. Fourth, further studies testing these effects with non-health behaviors might be useful to help understand the generalizability of the effects.

In summary, the present research shows cognitive-affective inconsistency and ambivalence to be strongly positively intercorrelated. It also shows cognitive-affective inconsistency and ambivalence to each be moderators of the overall attitude–behavior relationship (in individual tests) suggesting that both tap attitude strength. In addition, it shows that it is cognitive-affective inconsistency rather than ambivalence that moderates the overall attitude–behavior relationship when both are considered simultaneously. This suggests that such measures of inconsistency account for the effects of ambivalence on the overall attitude–behavior relationship. Testing whether cognitive-affective inconsistency also impacts on other aspects of attitude strength (e.g., attitude stability, pliability, and information processing; Krosnick & Petty, 1995) and explains the effects of cognitive-affective ambivalence may be a useful direction for future research.

## Supplemental Material

Conner_Online_Appendix – Supplemental material for Cognitive-Affective Inconsistency and Ambivalence: Impact on the Overall Attitude–Behavior Relationship Click here for additional data file. Supplemental material, Conner_Online_Appendix for Cognitive-Affective Inconsistency and Ambivalence: Impact on the Overall Attitude–Behavior Relationship by Mark Conner, Sarah Wilding, Frenk van Harreveld and Jonas Dalege in Personality and Social Psychology Bulletin
