# Supplementary material for: Cognitive-Affective Inconsistency and Ambivalence: Impact on the Overall Attitude–Behavior Relationship
Source: Pers Soc Psychol Bull. 2020 Aug 4;47(4):673–87. doi: 10.1177/0146167220945900 (PMC7961742; doi:10.1177/0146167220945900)
Supplement: Conner_Online_Appendix – Supplemental material for Cognitive-Affective Inconsistency and Ambivalence: Impact on the Overall Attitude–Behavior Relationship [file Conner_Online_Appendix.pdf]

Attitudes Towards Eating 5 Portions of Fruit and Vegetables Per Day

Thank you for replying to our advertisement. We are very pleased that you have agreed to participate in our research.

The study is being conducted as part of a larger project taking place both here at the University of Leeds and at the Institute of Food Research in Reading.

This study involves completing two questionnaires concerning your attitudes to eating a low fat diet. Each questionnaire should take approximately 20 minutes to complete. The questions in both questionnaires require you to tick the box or circle the number that best represents your own opinions or views. All your answers will be treated in the strictest confidence. The answers you provide will be stored on the computer to enable us to look at general trends, rather than individual responses. No individual responses will be reported. As required under the Data Protection Act (1984), your details held by us on computer are available to you on request.

You will be paid £5 for your participation in this research on the return of the second completed questionnaire. The address to which the questionnaires must be sent using the FREEPOST envelope provided is written on the final page of this questionnaire. You will receive the second questionnaire approximately two weeks after returning the first. In order that we may make payment to you, please fill in your name and address in the space provided below.

Your name (block capitals please)..

Your address (block capitals please)..

....

....

....

.... Postcode ....

Please note : You are under no obligation to take part in this study.

Please sign below to indicate that your participation is entirely voluntary.

Your signature: Date:

If you have any problems or enquiries, please phone Rachel Povey, on 0113-2335714.

Please turn over

Section 1 : General questions

First we would like to ask some questions about you. Please answer each question by placing a tick in the box which applies to you and/or writing in the appropriate spaces.

1. Are you FEMALE ☐ or MALE ☐ *(Tick one box)*

2. What is your date of birth? ______________________

3. What is your marital status? *(Tick one box)*

single	married/ living as married	divorced	separated	widowed
☐	☐	☐	☐	☐

4. Who lives with you in your household? *(Tick all that apply)*

no-one	your partner/spouse	your children	your friends	your parents	other *(please specify)*________
☐	☐	☐	☐	☐	☐

5. What is your ethnic origin? *(Please tick one box)*
   - ☐ White
   - ☐ Asian *Bangladeshi/Chinese/Indian/Pakistani/Other*
   - ☐ Black *African/Caribbean/Other*
   - ☐ Other ethnic group (please specify)

6. Which of the following qualifications do you have? *(Tick one box)*

none	'O' levels/ GCSE	'A' levels	HNC/	degree HND	higher degree	other *(please specify)*__________
☐	☐	☐	☐	☐	☐	☐

7. What is the occupation of the *main earner* in your household? *(if unemployed, please write their previous occupation)*______________________

8. What was your total household income over the past year?

£5499 or less	£5500-£9900	£9901-£1500	£15001-£21900	£21901 or more
☐	☐	☐	☐	☐

9. Do you follow a specific diet for medical or other reasons? YES ☐ NO ☐

10. If YES, what are these reasons? ______________________

____

Section 2 : Your Thoughts, Beliefs and Feelings about 5 Portions of Fruit and Vegetables per day

Health experts recommend that we should eat **five portions or servings of fruit and vegetables per day**. A portion is equivalent to, for example a large slice of melon, an apple, a bowl of salad, three serving spoons of peas or a glass of fruit juice. Foods which are not included are nuts, fruit drinks and squash, potatoes and processed foods (such as fruit cake and yoghurts).

The following questions concern your views and opinions about following this advice.

First list as many positive or negative **thoughts, beliefs** or **feelings** that you can think of with respect to eating 5 portions of fruit and vegetables per day. Write one in each box until no more come to mind.

Second, rate **each thought, belief** or **feeling** to indicate how positive or negative it is. For **extremely positive** thoughts/beliefs/feelings mark '+ + +' , for **very positive** mark '+ +' , for **slightly positive** mark '+'. If the thought/belief/feeling is **extremely negative** mark '- - -' , for **very negative** mark '- -', for **slightly negative** mark '-'. If the thought/belief/feeling is **neutral**, mark '**0**'.

The thoughts, beliefs or feelings I associate with **eating 5 portions of fruit and vegetables per day** are:		**Rating**
1.		______
2.		______
3.		______
4.		______
5.		______
6.		
7.		______
8.		______

Section 3: Your Positive Thoughts, Beliefs and Feelings about Eating 5 Portions of Fruit and Vegetables Per Day

1. Think about your *attitude toward or evaluation of* eating 5 portions of fruit and vegetables per day.

Considering only the favourable qualities of eating 5 portions of fruit and vegetables per day and ignoring the unfavourable characteristics, *how favourable is your evaluation of* eating 5 portions of fruit and vegetables per day? *Tick one box only*

Not at all favourable	Slightly favourable	Moderately favourable	Very favourable	Extremely favourable
☐	☐	☐	☐	☐

2. Think about your *feelings* toward eating 5 portions of fruit and vegetables per day.

Considering only your feelings about how enjoyable it would be to eat 5 portions of fruit and vegetables per day and ignoring your feelings of how unenjoyable it would be, *how enjoyable do you feel* eating 5 portions of fruit and vegetables per day would be? *Tick one box only*

Not at all enjoyable	Slightly enjoyable	Moderately enjoyable	Very enjoyable	Extremely enjoyable
☐	☐	☐	☐	☐

3. Think about your *thoughts* about eating 5 portions of fruit and vegetables per day.

Considering only the beneficial qualities of eating 5 portions of fruit and vegetables and ignoring the harmful characteristics, *how beneficial do you believe* eating 5 portions of fruit and vegetables to be? *Tick one box only*

Not at all beneficial	Slightly beneficial	Moderately beneficial	Very beneficial	Extremely beneficial
☐	☐	☐	☐	☐

4. Considering only the positive things about eating 5 portions of fruit and vegetables per day, and ignoring the negative things, *how positive are* those things? *Tick one box only*

Not at all positive	Slightly positive	Moderately positive	Very positive	Extremely positive
☐	☐	☐	☐	☐

Section 4 : Your Current Diet

Please read the questions below and tick one box (either "yes" or "no") for each question. If you have answered "yes" you may be required to answer a supplementary question.

1. Are you currently **in the process of changing to** eat 5 portions of fruit and vegetables per day?

☐ YES ☐ NO

If **YES**, for how long have you been making this change? *(Tick one box only)*

Less than a month	About 1-3 months	About 4-6 months	About 7-9 months	More than 10 months
☐	☐	☐	☐	☐

2. Are you currently **eating** 5 portions of fruit and vegetables per day? ☐ YES ☐ NO

3. Have you **changed** your diet to eat 5 portions of fruit and vegetables per day? ☐ YES ☐ NO

If **YES**, when did you change? *(Tick one box only)*

Less than a month ago	About 1-3 months ago	About 4-6 months ago	About 7-9 months ago	More than 10 months ago
☐	☐	☐	☐	☐

4. Are you currently **thinking about** whether or not you should eat 5 portions of fruit and vegetables per day in the future?

☐ YES ☐ NO

5. Have you **decided** to eat 5 portions of fruit and vegetables per day in the future?

☐ YES ☐ NO

If **YES**, when do you think it is most likely that you will begin to eat 5 portions of fruit and vegetables per day? *(Tick one box only)*

From now on	Beginning in the next few days	Beginning in the next few weeks	Beginning in about a month	Beginning in the next few months	Beginning in the distant future
☐	☐	☐	☐	☐	☐

6. Have you made any practical plans to eat 5 portions of fruit and vegetables per day in the future?

☐ YES ☐ NO

Section 5 : Your Negative Thoughts, Beliefs and Feelings about Eating 5 Portions of Fruit and Vegetables Per Day

1. Think about your *attitude toward or evaluation of* eating 5 portions of fruit and vegetables per day.

Considering only the unfavourable qualities of eating 5 portions of fruit and vegetables per day and ignoring the favourable characteristics, *how unfavourable is your evaluation* of eating 5 portions of fruit and vegetables per day? *Tick one box only*

Not at all unfavourable	Slightly unfavourable	Moderately unfavourable	Very unfavourable	Extremely unfavourable
☐	☐	☐	☐	☐

2. Think about your *feelings* toward eating 5 portions of fruit and vegetables per day.

Considering only your feelings of how unenjoyable it would be to eat 5 portions of fruit and vegetables per day and ignoring your feelings of how enjoyable it would be, *how unenjoyable do you feel* eating 5 portions of fruit and vegetables per day would be? *Tick one box only*

Not at all unenjoyable	Slightly unenjoyable	Quite unenjoyable	Moderately unenjoyable	Extremely unenjoyable
☐	☐	☐	☐	☐

3. Think about your *thoughts* about eating 5 portions of fruit and vegetables per day.

Considering only the harmful qualities of eating 5 portions of fruit and vegetables per day and ignoring the beneficial characteristics, *how harmful do you believe* eating 5 portions of fruit and vegetables to be?

Tick one box only

Not at all harmful	Slightly harmful	Moderately harmful	Very harmful	Extremely harmful
☐	☐	☐	☐	☐

4. Considering only the negative things about eating 5 portions of fruit and vegetables per day, and ignoring the positive things, *how negative are* those things?

Tick one box only

Not at all negative	Slightly negative	Moderately negative	Very negative	Extremely negative
☐	☐	☐	☐	☐

Section 6: Outcomes of Eating 5 Portions of Fruit and Vegetables per Day

This section asks questions concerning your thoughts about the outcomes of eating 5 portions of fruit and vegetables per day. There are no right or wrong answers, we are just interested in your point of view. In each case please *circle the number* which you feel most accurately represents your opinion.

1. Eating 5 portions of fruit and vegetables per day would give me a feeling of well-being.

Likely 1 2 3 4 5 6 7 **Unlikely**

2. Having a feeling of well-being would be...

Bad 1 2 3 4 5 6 7 **Good**

3. Eating 5 portions of fruit and vegetables per day would make me healthy.

Likely 1 2 3 4 5 6 7 **Unlikely**

4. Being healthy would be...

Bad 1 2 3 4 5 6 7 **Good**

5. Eating 5 portions of fruit and vegetables per day would be boring.

Likely 1 2 3 4 5 6 7 **Unlikely**

6. A boring diet would be...

Bad 1 2 3 4 5 6 7 **Good**

7. Eating 5 portions of fruit and vegetables per day would mean missing out on food I like.

Likely 1 2 3 4 5 6 7 **Unlikely**

8. Missing out on food I like would be...

Bad 1 2 3 4 5 6 7 **Good**

9. Eating 5 portions of fruit and vegetables per day would make me feel energetic.

Likely 1 2 3 4 5 6 7 **Unlikely**

10. Feeling energetic would be...

Bad 1 2 3 4 5 6 7 **Good**

11. Eating 5 portions of fruit and vegetables per day would reduce my weight.

Likely 1 2 3 4 5 6 7 **Unlikely**

12. Reducing my weight would be...

Bad 1 2 3 4 5 6 7 **Good**

13. Eating 5 portions of fruit and vegetables per day would be tasteless.

Likely 1 2 3 4 5 6 7 **Unlikely**

14. A tasteless diet would be...

Bad 1 2 3 4 5 6 7 **Good**

15. Eating 5 portions of fruit and vegetables per day would give me a healthy complexion.

Likely 1 2 3 4 5 6 7 **Unlikely**

16. Having a healthy complexion would be...

Bad 1 2 3 4 5 6 7 **Good**

17. By eating 5 portions of fruit and vegetables per day, I would reduce my risk of getting heart disease.

Likely 1 2 3 4 5 6 7 **Unlikely**

18. Reducing my risk of getting heart disease would be...

Bad 1 2 3 4 5 6 7 **Good**

19. By eating 5 portions of fruit and vegetables per day, I would reduce my risk of getting cancer.

Likely 1 2 3 4 5 6 7 **Unlikely**

20. Reducing my risk of getting cancer would be...

Bad 1 2 3 4 5 6 7 **Good**

Section 7 : Views and Opinions of People You Know

This section is about what other people you know think of you eating 5 portions of fruit and vegetables per day. In each case please *circle the number* which you feel most accurately represents your opinion.

1. Most of the people I know eat 5 portions of fruit and vegetables per day.

Strongly agree 1 2 3 4 5 6 7 **Strongly disagree**

2. People who are important to me think I...

Should eat 5 portions of fruit and vegetables per day 1 2 3 4 5 6 7 **Should not eat 5 portions of fruit and vegetables per day**

3. People who are important to me would...

Disapprove of my eating 5 portions of fruit and vegetables per day 1 2 3 4 5 6 7 **Approve of my eating 5 portions of fruit and vegetables per day**

4. People who are important to me want me to eat 5 portions of fruit and vegetables per day.

Strongly agree 1 2 3 4 5 6 7 **Strongly disagree**

5. I feel under social pressure to eat 5 portions of fruit and vegetables per day.

Strongly agree 1 2 3 4 5 6 7 **Strongly disagree**

6. People who are important to me influence my decision to eat 5 portions of fruit and vegetables per day

Strongly agree 1 2 3 4 5 6 7 **Strongly disagree**

7. My work colleagues think I...

Should eat 5 portions of fruit and vegetables per day 1 2 3 4 5 6 7 **Should not eat 5 portions of fruit and vegetables per day**

8. With regard to eating 5 portions of fruit and vegetables per day, how much do you want to do what your work colleagues think you should?

Very much 1 2 3 4 5 6 7 **Not at all**

9. My doctor probably thinks I...

**Should eat 5 portions of fruit and vegetables per day**	1 2 3 4 5 6 7	**Should not eat 5 portions of fruit and vegetables per day**

10. With regard to eating 5 portions of fruit and vegetables per day, how much do you want to do what your doctor thinks you should?

Very much 1 2 3 4 5 6 7 **Not at all**

11. My friends think I...

**Should not eat 5 portions of fruit and vegetables per day**	1 2 3 4 5 6 7	**Should eat 5 portions of fruit and vegetables per day**

12. With regard to eating 5 portions of fruit and vegetables per day, how much do you want to do what your friends think you should?

Not at all 1 2 3 4 5 6 7 **Very much**

13. The people I live with think I...

**Should not eat 5 portions of fruit and vegetables per day**	1 2 3 4 5 6 7	**Should eat 5 portions of fruit and vegetables per day**

14. With regard to eating 5 portions of fruit and vegetables per day, how much do you want to do what the people you live with think you should?

Not at all 1 2 3 4 5 6 7 **Very much**

15. My parents think I...

**Should not eat 5 portions of fruit and vegetables per day**	1 2 3 4 5 6 7	**Should eat 5 portions of fruit and vegetables per day**

16. With regard to eating 5 portions of fruit and vegetables per day, how much do you want to do what your parents think you should?

Not at all 1 2 3 4 5 6 7 **Very much**

Section 8: Helps & Hindrances to Eating 5 Portions of Fruit & Vegetables per Day

The following questions refer to helps and hindrances that some people have mentioned about eating 5 portions of fruit and vegetables per day. For each question, please *circle the number* which you feel most accurately represents your opinion.

1. For me, eating 5 portions of fruit and vegetables per day would be time consuming.

Strongly agree 1 2 3 4 5 6 7 **Strongly disagree**

2. Being time consuming to eat 5 portions of fruit and vegetables per day would make my eating them...

Extremely easy 1 2 3 4 5 6 7 **Extremely difficult**

3. For me, eating 5 portions of fruit and vegetables per day would not cost much money.

Strongly agree 1 2 3 4 5 6 7 **Strongly disagree**

4. Not costing much money to eat 5 portions of fruit and vegetables per day would make my eating them...

Extremely easy 1 2 3 4 5 6 7 **Extremely difficult**

5. For me, eating 5 portions of fruit and vegetables per day would require a lot of preparation.

Strongly agree 1 2 3 4 5 6 7 **Strongly disagree**

6. Requiring a lot of preparation to eat 5 portions of fruit and vegetables per day would make my eating them...

Extremely easy 1 2 3 4 5 6 7 **Extremely difficult**

7. For me, eating 5 portions of fruit and vegetables per day would require strong motivation

Strongly agree 1 2 3 4 5 6 7 **Strongly disagree**

8. Requiring a strong motivation to eat 5 portions of fruit and vegetables per day would make my eating them...

Extremely easy 1 2 3 4 5 6 7 **Extremely difficult**

9. The people around me eat 5 portions of fruit and vegetables per day.

Strongly agree 1 2 3 4 5 6 7 **Strongly disagree**

10. People around me eating 5 portions of fruit and vegetables per day would make my eating them...

Extremely easy 1 2 3 4 5 6 7 **Extremely difficult**

11. I don't have enough knowledge about eating 5 portions of fruit and vegetables per day.

Strongly agree 1 2 3 4 5 6 7 **Strongly disagree**

12. Not having enough knowledge about eating 5 portions of fruit and vegetables per day would make my eating them...

Extremely easy 1 2 3 4 5 6 7 **Extremely difficult**

13. For me, fruit and vegetables are readily available.

Strongly agree 1 2 3 4 5 6 7 **Strongly disagree**

14. Fruit and vegetables being readily available would make my eating 5 portions of fruit and vegetables per day...

Extremely easy 1 2 3 4 5 6 7 **Extremely difficult**

15. Fruit and vegetables do not store well.

Strongly agree 1 2 3 4 5 6 7 **Strongly disagree**

16. Fruit and vegetables not storing well would make my eating 5 portions of fruit and vegetables per day...

Extremely easy 1 2 3 4 5 6 7 **Extremely difficult**

17. To what extent do you feel that you <u>need</u> to eat 5 portions of fruit and vegetables per day?

Not at all 1 2 3 4 5 6 7 **To an extremely great extent**

18. I *intend* to eat 5 portions of fruit and vegetables per day.

Definitely do not 1 2 3 4 5 6 7 **Definitely do**

19. I *plan* to eat 5 portions of fruit and vegetables per day.

Definitely do not 1 2 3 4 5 6 7 **Definitely do**

20. I *want* to eat 5 portions of fruit and vegetables per day.

Definitely do not 1 2 3 4 5 6 7 **Definitely do**

21. If I were to eat 5 portions of fruit and vegetables per day, it would be:

Circle one number per line

**Bad**	1 2 3 4 5 6 7	**Good**
**Beneficial**	1 2 3 4 5 6 7	**Harmful**
**Unpleasant**	1 2 3 4 5 6 7	**Pleasant**
**Favourable**	1 2 3 4 5 6 7	**Unfavourable**
**Positive**	1 2 3 4 5 6 7	**Negative**
**Satisfactory**	1 2 3 4 5 6 7	**Unsatisfactory**
**Enjoyable**	1 2 3 4 5 6 7	**Unenjoyable**

22. It is mostly up to me whether or not I eat 5 portions of fruit and vegetables per day from now on.

Strongly agree 1 2 3 4 5 6 7 **Strongly disagree**

23. If I wanted to, I could easily eat 5 portions of fruit and vegetables per day from now on.

Extremely likely 1 2 3 4 5 6 7 **Extremely unlikely**

24. For me, eating 5 portions of fruit and vegetables per day would be...

Easy 1 2 3 4 5 6 7 **Difficult**

25. How much control do you have over eating 5 portions of fruit and vegetables per day from now on?

Complete control 1 2 3 4 5 6 7 **Absolutely no control**

26. What is the likelihood that if you tried you would be able to eat 5 portions of fruit and vegetables per day from now on?

Likely 1 2 3 4 5 6 7 **Unlikely**

27. How much personal control do you feel you would have over whether or not you eat 5 portions of fruit and vegetables per day from now on?

Complete control 1 2 3 4 5 6 7 **No control**

28. How certain are you that you could eat 5 portions of fruit and vegetables per day from now on?

Not at all certain 1 2 3 4 5 6 7 **Very certain**

29. The number of events outside my control which could prevent me from eating 5 portions of fruit and vegetables per day from now on are... ?

Numerous 1 2 3 4 5 6 7 **Very few**

30. How much control do you have over whether you do or do not eat 5 portions of fruit and vegetables per day from now on?

Complete control 1 2 3 4 5 6 7 **Very little control**

31. For me to eat 5 portions of fruit and vegetables per day from now on would be...

Extremely easy 1 2 3 4 5 6 7 **Extremely difficult**

Thankyou for your help in completing this questionnaire
Please return this questionnaire using the FREEPOST envelope provided.
If you have any comments, problems or queries, please contact Rachel Povey, on 0113-2335714.

64

Attitudes Towards Low Fat Diets

Thank you for replying to our advertisement. We are very pleased that you have agreed to participate in our research.

The study is being conducted as part of a larger project taking place both here at the University of Leeds and at the Institute of Food Research in Reading.

This study involves completing two questionnaires concerning your attitudes to eating a low fat diet. Each questionnaire should take approximately 20 minutes to complete. The questions in both questionnaires require you to tick the box or circle the number that best represents your own opinions or views. All your answers will be treated in the strictest confidence. The answers you provide will be stored on the computer to enable us to look at general trends, rather than individual responses. No individual responses will be reported. As required under the Data Protection Act (1984), your details held by us on computer are available to you on request.

You will be paid £5 for your participation in this research on the return of the second completed questionnaire. The address to which the questionnaires must be sent using the FREEPOST envelope provided is written on the final page of this questionnaire. You will receive the second questionnaire approximately two weeks after returning the first. In order that we may make payment to you, please fill in your name and address in the space provided below.

Your name (block capitals please) .... [illegible]

Your address (block capitals please) .... [illegible]

....

....

....

.... Postcode B3 3TB

Please note : You are under no obligation to take part in this study.

Please sign below to indicate that your participation is entirely voluntary.

Your signature: [illegible] Date: 1/4/96

If you have any problems or enquiries, please phone Rachel Povey, on 0113-2335714.

Please turn over

64

Attitudes Towards Low Fat Diets

Thank you for replying to our advertisement. We are very pleased that you have agreed to participate in our research.

The study is being conducted as part of a larger project taking place both here at the University of Leeds and at the Institute of Food Research in Reading.

This study involves completing two questionnaires concerning your attitudes to eating a low fat diet. Each questionnaire should take approximately 20 minutes to complete. The questions in both questionnaires require you to tick the box or circle the number that best represents your own opinions or views. All your answers will be treated in the strictest confidence. The answers you provide will be stored on the computer to enable us to look at general trends, rather than individual responses. No individual responses will be reported. As required under the Data Protection Act (1984), your details held by us on computer are available to you on request.

You will be paid £5 for your participation in this research on the return of the second completed questionnaire. The address to which the questionnaires must be sent using the FREEPOST envelope provided is written on the final page of this questionnaire. You will receive the second questionnaire approximately two weeks after returning the first. In order that we may make payment to you, please fill in your name and address in the space provided below.

Your name (block capitals please) .... [illegible]

Your address (block capitals please) .... [illegible]

.... [illegible]

.... [illegible]

....

.... Postcode [illegible]

Please note : You are under no obligation to take part in this study.

Please sign below to indicate that your participation is entirely voluntary.

Your signature: [illegible] Date: 1/4/96

If you have any problems or enquiries, please phone Rachel Povey, on 0113-2335714.

Please turn over

04

Section 1 : General questions

First we would like to ask some questions about you. Please answer each question by placing a tick in the box which applies to you and/or writing in the appropriate spaces.

1. Are you FEMALE ☑ or MALE ☐ *(Tick one box)*

2. What is your date of birth? 30·12·71

3. What is your marital status? *(Tick one box)*

single	married/ living as married	divorced	separated	widowed
☑	☐	☐	☐	☐

4. Who lives with you in your household? *(Tick all that apply)*

no-one	your partner/spouse	your children	your friends	your parents	other *(please specify)*
☐	☑	☐	☐	☐	☐

5. What is your ethnic origin? *(Please tick one box)*

☐ White
☑ Asian *~~Bangladeshi~~/Chinese/~~Indian/Pakistani/Other~~*
☐ Black *African/Caribbean/Other*
☐ Other ethnic group (please specify)

6. Which of the following qualifications do you have? *(Tick one box)*

none	'O' levels/	'A' levels GCSE	HNC/	degree HND	higher degree	other *(please specify)*
☐	☐	☐	☐	☑	☐	☐

7. What is the occupation of the *main earner* in your household? *(if unemployed, please write their previous occupation)* Housing Office

8. What was your total household income over the past year?

£5499 or less	£5500-£9900	£9901-£1500	£15001-£21900	£21901 or more
☐	☑	☐	☐	☐

9. Do you follow a specific diet for medical or other reasons? YES ☐ NO ☑

10. If YES, what are these reasons? ________________

Section 2 : Your Thoughts, Beliefs and Feelings about Eating a Low Fat Diet

Health experts recommend that we should eat a **low fat diet**. Specifically, they recommend that fat should provide **no more than 35%** of the food energy in our diet.

The following questions concern your views and opinions about following this advice.

First, list as many positive or negative **thoughts, beliefs** or **feelings** that you can think of with respect to eating a low fat diet. Write one in each box until no more come to mind.

Second, rate **each thought, belief** or **feeling** to indicate how positive or negative it is. For **extremely positive** thoughts/beliefs/feelings mark '+ + +', for **very positive** mark '+ +', for **slightly positive** mark '+'. If the thought/belief/feeling is **extremely negative** mark '- - -', for **very negative** mark '- -', for **slightly negative** mark '-'. If the thought/belief/feeling is **neutral**, mark '0'.

The thoughts, beliefs or feelings I associate with **eating a low fat diet** are:	**Rating**
1. Good for health	+++
2. help to lose weight	+++
3. less tasty foods	++
4.	
5.	
6.	
7.	
8.	

Section 3: Your Positive Thoughts, Beliefs and Feelings about Eating a Low Fat Diet

1. Think about your *attitude toward or evaluation of* eating a low fat diet.

Considering only the favourable qualities of eating a low fat diet and ignoring the unfavourable characteristics, *how favourable is your evaluation of* eating a low fat diet? *Tick one box only*

Not at all favourable	Slightly favourable	Moderately favourable	Very favourable	Extremely favourable
☐	☐	☐	☐	☐ ✓

2. Think about your *feelings* toward eating a low fat diet.

Considering only your feelings about how enjoyable it would be to eat a low fat diet and ignoring your feelings of how unenjoyable it would be, *how enjoyable do you feel* eating a low fat diet would be? *Tick one box only*

Not at all enjoyable	Slightly enjoyable	Moderately enjoyable	Very enjoyable	Extremely enjoyable
☐	☐	☐	☐ ✓	☐

3. Think about your *thoughts* about eating a low fat diet.

Considering only the beneficial qualities of eating a low fat diet and ignoring the harmful characteristics, *how beneficial do you believe* eating a low fat diet to be? *Tick one box only*

Not at all beneficial	Slightly beneficial	Moderately beneficial	Very beneficial	Extremely beneficial
☐	☐	☐	☐	☐ ✓

4. Considering only the positive things about eating a low fat diet, and ignoring the negative things, *how positive are* those things? *Tick one box only*

Not at all positive	Slightly positive	Moderately positive	Very positive	Extremely positive
☐	☐	☐	☐ ✓	☐

Section 4 : Your Current Diet

Please read the questions below and tick one box (either "yes" or "no") for each question. If you have answered "yes" you may be required to answer a supplementary question.

1. Are you currently **in the process of changing to** a low fat diet? ☐ YES ☐ ✓ NO

If **YES**, for how long have you been making this change? *Tick one box only*

Less than a month	About 1-3 months	About 4-6 months	About 7-9 months	More than 10 months
☐	☐	☐	☐	☐

2. Are you **currently eating** a low fat diet? ☐ ✓ YES ☐ NO

3. Have you **changed** your diet to eat a low fat diet? ☑ YES ☐ NO

If **YES**, when did you change? *Tick one box only*

Less than a month ago	About 1-3 months ago	About 4-6 months ago	About 7-9 months ago	More than 10 months ago
☐	☐	☐	☐	☑

4. Are you currently **thinking about** whether or not you should eat a low fat diet in the future?

☐ YES ☑ NO

5. Have you **decided** to eat a low fat diet in the future? ☑ YES ☐ NO

If **YES**, when do you think it is most likely that you will begin to eat a low fat diet? *Tick one box only*

From now on	Beginning in the next few days	Beginning in the next few weeks	Beginning in about a month	Beginning in the next few months	Beginning inthe distant future
☑	☐	☐	☐	☐	☐

6. Have you made any practical plans to eat a low fat diet in the future? ☑ YES ☐ NO

Section 5 : Your Negative Thoughts, Beliefs and Feelings about Eating a Low Fat Diet

1. Think about your *attitude toward or evaluation of* eating a low fat diet.

Considering only the unfavourable qualities of eating a low fat diet and ignoring the favourable characteristics, *how unfavourable is your evaluation* of eating a low fat diet? *Tick one box only*

Not at all unfavourable	Slightly unfavourable	Moderately unfavourable	Very unfavourable	Extremely unfavourable
☐	☑	☐	☐	☐

2. Think about your *feelings* toward eating a low fat diet.

Considering only your feelings of how unenjoyable it would be to eat a low fat diet and ignoring your feelings of how enjoyable it would be, *how unenjoyable do you feel* eating a low fat diet would be? *Tick one box only*

Not at all unenjoyable	Slightly unenjoyable	Quite unenjoyable	Moderately unenjoyable	Extremely unenjoyable
☐	☐	☐	☑	☐

3. Think about your *thoughts* about eating a low fat diet.

Considering only the harmful qualities of eating a low fat diet and ignoring the beneficial characteristics, *how harmful do you believe* eating a low fat diet to be? *Tick one box only*

Not at all harmful	Slightly harmful	Moderately harmful	Very harmful	Extremely harmful
☑	☐	☐	☐	☐

4. Considering only the negative things about eating a low fat diet, and ignoring the positive things, *how negative are* those things? *Tick one box only*

Not at all negative	Slightly negative	Moderately negative	Very negative	Extremely negative
☑	☐	☐	☐	☐

Section 6: Outcomes of Eating a Low Fat Diet

This section asks questions concerning your thoughts about the outcomes of eating a low fat diet. There are no right or wrong answers, we are just interested in your point of view. In each case please *circle the number* which you feel most accurately represents your opinion.

1. Eating a low fat diet would give me a feeling of well-being.

Likely 1 (2) 3 4 5 6 7 **Unlikely**

2. Having a feeling of well-being would be...

Bad 1 2 3 4 5 6 (7) **Good**

3. Eating a low fat diet would make me healthy.

Likely (1) 2 3 4 5 6 7 **Unlikely**

4. Being healthy would be...

Bad 1 2 3 4 5 6 (7) **Good**

5. Eating a low fat diet would be boring.

Likely 1 2 (3) 4 5 6 7 **Unlikely**

6. A boring diet would be...

Bad 1 2 (3) 4 5 6 7 **Good**

7. Eating a low fat diet would mean missing out on food I like.

Likely (1) 2 3 4 5 6 7 **Unlikely**

8. Missing out on food I like would be...

Bad 1 2 (3) 4 5 6 7 **Good**

9. Eating a low fat diet would make me feel energetic.

Likely 1 (2) 3 4 5 6 7 **Unlikely**

10. Feeling energetic would be...

Bad 1 2 3 4 5 6 (7) **Good**

11. Eating a low fat diet would reduce my weight.

Likely 1 (2) 3 4 5 6 7 **Unlikely**

12. Reducing my weight would be...

Bad 1 2 3 4 5 6 (7) **Good**

13. Eating a low fat diet would be tasteless.

Likely 1 2 (3) 4 5 6 7 **Unlikely**

14. A tasteless diet would be...

Bad (1) 2 3 4 5 6 7 **Good**

15. Eating a low fat diet would give me a healthy complexion.

Likely (1) 2 3 4 5 6 7 **Unlikely**

16. Having a healthy complexion would be...

Bad 1 2 3 4 5 6 (7) **Good**

17. By eating a low fat diet, I would reduce my risk of getting heart disease.

Likely (1) 2 3 4 5 6 7 **Unlikely**

18. Reducing my risk of getting heart disease would be...

Bad 1 2 3 4 5 (6) 7 **Good**

19. By eating a low fat diet, I would reduce my risk of getting cancer.

Likely 1 (2) 3 4 5 6 7 **Unlikely**

20. Reducing my risk of getting cancer would be...

Bad 1 2 3 4 5 (6) 7 **Good**

Section 7 : Views and Opinions of People You Know

This section is about what other people you know think of you eating a low fat diet. In each case please *circle the number* which you feel most accurately represents your opinion.

1. Most of the people I know eat a low fat diet.

Strongly agree 1 2 3 4 5 6 7 **Strongly disagree**

2. People who are important to me think I...

Should eat a low fat diet 1 2 3 4 5 6 7 **Should not eat a low fat diet**

3. People who are important to me would...

Disapprove of my eating a low fat diet 1 2 3 4 5 6 7 **Approve of my eating a low fat diet**

4. People who are important to me want me to eat a low fat diet.

Strongly agree 1 2 3 4 5 6 7 **Strongly disagree**

5. I feel under social pressure to eat a low fat diet.

Strongly agree 1 2 3 4 5 6 7 **Strongly disagree**

6. People who are important to me influence my decision to eat a low fat diet

Strongly agree 1 2 3 4 5 6 7 **Strongly disagree**

7. My work colleagues think I...

Should eat a low fat diet 1 2 3 4 5 6 7 **Should not eat a low fat diet**

8. With regard to eating a low fat diet, how much do you want to do what your work colleagues think you should?

Very much 1 2 3 4 5 6 7 **Not at all**

9. My doctor probably thinks I...

Should eat a low fat diet 1 2 3 4 5 6 7 **Should not eat a low fat diet**

10. With regard to eating a low fat diet, how much do you want to do what your doctor thinks you should?

Very much 1 2 3 4 5 6 7 **Not at all**

11. My friends think I...

Should not eat a low fat diet 1 2 3 (4) 5 6 7 **Should eat a low fat diet**

12. With regard to eating a low fat diet, how much do you want to do what your friends think you should?

Not at all 1 2 3 4 (5) 6 7 **Very much**

13. The people I live with think I...

Should not eat a low fat diet 1 2 3 4 (5) 6 7 **Should eat a low fat diet**

14. With regard to eating a low fat diet, how much do you want to do what the people you live with think you should?

Not at all 1 2 3 4 (5) 6 7 **Very much**

15. My family thinks I...

Should not eat a low fat diet 1 2 3 (4) 5 6 7 **Should eat a low fat diet**

16. With regard to eating a low fat diet, how much do you want to do what your family thinks you should?

Not at all 1 2 3 (4) 5 6 7 **Very much**

Section 8: Helps and Hindrances to Eating a Low Fat Diet

The following questions refer to helps and hindrances that some people have mentioned about eating a low fat diet. For each question, please *circle the number* which you feel most accurately represents your opinion.

1. For me, a low fat diet would be time consuming.

Strongly agree 1 2 (3) 4 5 6 7 **Strongly disagree**

2. A low fat diet which is time consuming would make eating a low fat diet...

Extremely easy 1 2 3 4 (5) 6 7 **Extremely difficult**

3. For me, a low fat diet would not cost much money.

Strongly agree 1 2 3 4 (5) 6 7 **Strongly disagree**

4. A low fat diet which does not cost much money would make eating a low fat diet...

Extremely easy 1 2 3 4 5 6 7 **Extremely difficult**

5. For me, a low fat diet would require a lot of preparation.

Strongly agree 1 2 3 4 5 6 7 **Strongly disagree**

6. A low fat diet which requires a lot of preparation would make eating a low fat diet...

Extremely easy 1 2 3 4 5 6 7 **Extremely difficult**

7. For me, eating a low fat diet would require strong motivation

Strongly agree 1 2 3 4 5 6 7 **Strongly disagree**

8. A low fat diet which requires strong motivation would make eating a low fat diet...

Extremely easy 1 2 3 4 5 6 7 **Extremely difficult**

9. The people around me eat a low fat diet.

Strongly agree 1 2 3 4 5 6 7 **Strongly disagree**

10. People around me eating a low fat diet would make eating a low fat diet...

Extremely easy 1 2 3 4 5 6 7 **Extremely difficult**

11. I don't have enough knowledge about low fat diets.

Strongly agree 1 2 3 4 5 6 7 **Strongly disagree**

12. Not having enough knowledge about low fat diets would make eating a low fat diet...

Extremely easy 1 2 3 4 5 6 7 **Extremely difficult**

13. For me, low fat foods are readily available.

Strongly agree 1 2 3 4 5 6 7 **Strongly disagree**

14. Low fat foods being readily available would make my eating a low fat diet...

Extremely easy 1 2 3 4 5 6 7 **Extremely difficult**

15. Low fat foods do not store well.

Strongly agree 1 2 3 4 5 6 7 **Strongly disagree**

16. Low fat foods not storing well would make my eating a low fat diet...

Extremely easy 1 2 3 4 5 6 7 **Extremely difficult**

17. To what extent do you feel that you <u>need</u> to eat a low fat diet?

Not at all 1 2 3 4 5 6 7 **To an extremely great extent**

18. I *intend* to eat a low fat diet.

Definitely do not 1 2 3 4 5 6 7 **Definitely do**

19. I *plan* to eat a low fat diet.

Definitely do not 1 2 3 4 5 6 7 **Definitely do**

20. I *want* to eat a low fat diet.

Definitely do not 1 2 3 4 5 6 7 **Definitely do**

21. If I were to eat a low fat diet, it would be: *Circle one number per line*

**Bad**	1 2 3 4 5 6 7	**Good**
**Beneficial**	1 2 3 4 5 6 7	**Harmful**
**Unpleasant**	1 2 3 4 5 6 7	**Pleasant**
**Favourable**	1 2 3 4 5 6 7	**Unfavourable**
**Positive**	1 2 3 4 5 6 7	**Negative**
**Satisfactory**	1 2 3 4 5 6 7	**Unsatisfactory**
**Enjoyable**	1 2 3 4 5 6 7	**Unenjoyable**

22. It is mostly up to me whether or not I eat a low fat diet from now on.

Strongly agree 1 2 3 4 5 6 7 **Strongly disagree**

23. If I wanted to, I could easily eat a low fat diet from now on.

Extremely likely 1 2 3 4 5 6 7 **Extremely unlikely**

24. For me, eating a low fat diet would be...

Easy 1 2 3 4 5 6 7 **Difficult**

25. How much control do you have over eating a low fat diet from now on?

Complete control 1 2 3 4 5 6 7 **Absolutely no control**

26. What is the likelihood that if you tried you would be able to eat a low fat diet from now on?

Likely 1 2 3 4 5 6 7 **Unlikely**

27. How much personal control do you feel you would have over whether or not you eat a low fat diet from now on?

Complete control 1 2 3 4 5 6 7 **No control**

28. How certain are you that you could eat a low fat diet from now on?

Not at all certain 1 2 3 4 5 6 7 **Very certain**

29. The number of events outside my control which could prevent me from eating a low fat diet from now on are...

Numerous 1 2 3 4 5 6 7 **Very few**

30. How much control do you have over whether you do or do not eat a low fat diet from now on?

Complete control 1 2 3 4 5 6 7 **Very little control**

31. For me to eat a low fat diet from now on would be...

Extremely easy 1 2 3 4 5 6 7 **Extremely difficult**

Thankyou for your help in completing this questionnaire

If you have any additional comments to make, please write them in the box below.

Please return this questionnaire using the FREEPOST envelope provided.

If you have any problems or queries, please contact Rachel Povey, on 0113-2335714.

Attitudes Towards Low Fat Diets: Part 2

Thankyou for completing Part 1 of this questionnaire.

Part 2 of the questionnaire is concerned with your views and opinions about your current diet, and also about the content of your current diet. For each question, please tick the most appropriate box or circle the number that best represents your own opinions or views. Once again, all answers will be treated in the strictest confidence. The answers that you provide will be stored as codes on the computer to enable us to look at general trends, rather than individual responses. No individual responses will be reported. As required under the Data Protection Act (1984), your details held by us on computer are available to you on request.

When you have completed this questionnaire, please return using the FREEPOST envelope provided to : Rachel Povey, Attitudes Research Project, Health Promotion Research Group, University of Leeds, FREEPOST LS3018, Leeds, LS2 1YY.

On receipt of this completed questionnaire, we will send you a cheque for £5. Please allow 21 days for receipt of your cheque. Please write below the name to which you would like the cheque to be written out to, your name (if different), and your address:

Cheque to be made payable to (block capitals please)..

Your name and address (block capitals please)...

....

....

....Postcode ....

Signature: Date:

Thankyou again for participating in this research.

Yours sincerely,

Rachel Povey
Research Assistant
Health Promotion Research Group
University of Leeds

Please turn over

Section 1 : Views about your current diet

The following questions concern your views and opinions about your **current diet**. There are no right or wrong answers, we are just interested in your point of view. In each case, please tick the box or circle the number which you feel most accurately describes your opinion.

1. I would describe my diet as: *(Circle one number per line)*

**Bad**	1	2	3	4	5	6	7	**Good**
**Beneficial**	1	2	3	4	5	6	7	**Harmful**
**Unpleasant**	1	2	3	4	5	6	7	**Pleasant**
**Favourable**	1	2	3	4	5	6	7	**Unfavourable**
**Negative**	1	2	3	4	5	6	7	**Positive**
**Satisfactory**	1	2	3	4	5	6	7	**Unsatisfactory**
**Enjoyable**	1	2	3	4	5	6	7	**Unenjoyable**

2. Do you think your current diet is : *(Tick one box only)*

Very healthy	Healthy	Slightly healthy	Neither healthy nor unhealthy	Slightly unhealthy	Unhealthy	Very unhealthy
☐	☐	☐	☐	☐	☐	☐

3. Would you say the fat content in your current diet is: *(Tick one box only)*

Very low	Low	Fairly low	Medium	Fairly high	High	Very high
☐	☐	☐	☐	☐	☐	☐

4. Would you say the fruit content in your current diet is: *(Tick one box only)*

Very low	Low	Fairly low	Medium	Fairly high	High	Very high
☐	☐	☐	☐	☐	☐	☐

5. Would you say the vegetable content in your current diet is: *(Tick one box only)*

Very low	Low	Fairly low	Medium	Fairly high	High	Very high
☐	☐	☐	☐	☐	☐	☐

6. Would you say the fibre content in your current diet is: *(Tick one box only)*

Very low	Low	Fairly low	Medium	Fairly high	High	Very high
☐	☐	☐	☐	☐	☐	☐

7. I am eating a low fat diet at the moment.

Strongly agree 1 2 3 4 5 6 7 **Strongly disagree**

8. I have tried to eat a low fat diet in the past month.

Strongly agree 1 2 3 4 5 6 7 **Strongly disagree**

9. I tend to eat foods that are low in fat:

Strongly agree 1 2 3 4 5 6 7 **Strongly disagree**

10. I tend to eat foods that are high in fat:

Strongly agree 1 2 3 4 5 6 7 **Strongly disagree**

11. Compared to an average person of your age and sex in the U.K., would you say that you:

Eat much more fat 1 2 3 4 5 6 7 **Eat much less fat**

12. To what extent do you believe your fat intake corresponds to the dietary recommendations i.e. that fat provides no more than 35% of the food energy in your diet?

Not at all 1 2 3 4 5 6 7 **To a very great extent**

13. I think of myself as a "healthy eater".

Strongly agree 1 2 3 4 5 6 7 **Strongly disagree**

14. I think of myself as someone who is concerned with healthy eating.

Strongly agree 1 2 3 4 5 6 7 **Strongly disagree**

15. I think of myself as someone who is concerned about the health consequences of what I eat.

Strongly agree 1 2 3 4 5 6 7 **Strongly disagree**

16. I think of myself as someone who enjoys the pleasures of eating.

Strongly agree 1 2 3 4 5 6 7 **Strongly disagree**

Please turn over

Section 2 : Your Eating Habits

The following questions concern your eating habits. In each case, please tick the box or circle the number which you feel most accurately describes your opinion.

1. For the following questions, if the question does not apply to the way you eat, tick **"not applicable"**.

In the **PAST MONTH**, how often did you...	Usually or Always	Often	Sometimes	Rarely or Never	**Not Applicable**
a) When eating chicken, have it baked or boiled?	☐	☐	☐	☐	☐
b) When eating chicken, take off the skin?	☐	☐	☐	☐	☐
c) Use a meatless tomato sauce on spaghetti or noodles?	☐	☐	☐	☐	☐
d) When eating red meat, eat only small portions?	☐	☐	☐	☐	☐
e) When eating red meat, cut off all visible fat?	☐	☐	☐	☐	☐
f) Have a vegetarian dinner?	☐	☐	☐	☐	☐
g) Eat fish or chicken instead of red meat?	☐	☐	☐	☐	☐
h) Use skimmed milk?	☐	☐	☐	☐	☐
i) Eat low-fat or diet cheese?	☐	☐	☐	☐	☐
j) Put butter or margarine on cooked vegetables?	☐	☐	☐	☐	☐
k) Eat boiled or baked potatoes without butter or margarine?	☐	☐	☐	☐	☐
l) Use low-calorie instead of regular salad dressing?	☐	☐	☐	☐	☐
m) Have only fruit for dessert?	☐	☐	☐	☐	☐
n) Eat at least two vegetables (not green salad) at dinner?	☐	☐	☐	☐	☐
o) Snack on raw vegetables instead of crisps?	☐	☐	☐	☐	☐
p) Eat bread or rolls without butter or margarine?	☐	☐	☐	☐	☐

2. How often do you use dairy products?

Never 1 2 3 4 5 6 7 **Frequently**

3. How often do you choose low fat alternatives (such as low fat yoghurt, or low fat spreads?)

Never 1 2 3 4 5 6 7 **Always**

4. How often do you eat snacks such as crisps, chocolate, cakes, pastries and biscuits?

Never 1 2 3 4 5 6 7 **Frequently**

5. How often do you fry foods?

Never 1 2 3 4 5 6 7 **Frequently**

6. How often do you replace meat with vegetables, potatoes or pulses?

Never 1 2 3 4 5 6 7 **Always**

7. In the following section, please answer **ALL** the questions. Select the answer that best applies to your situation and TICK the appropriate column. Only tick **one** box on **each** line (i.e., one tick per food). If you make a mistake, put a cross through the incorrect tick and then tick the correct answer.

For example: **How often do you eat the following foods?** (tick the appropriate box on each line)

		2 or more times a day	Every day	3-5 times a week	1-2 times a week	1-3 times a month	Rarely-never
Milk (including in tea or coffee)	Whole	☑	☐	☐	☐	☐	☐
	Semi- skimmed	☐	☐	☐	☒	☐	☑
	Skimmed	☐	☐	☐	☐	☑	☐

How often do you eat each of the following foods? (tick the appropriate box on each line)

Please record what you normally eat and drink:

	2 or more times a day	Every day	3-5 times a week	1-2 times a week	1-3 times a month	Rarely-never
Milk (including in tea/coffee) :Whole	☐	☐	☐	☐	☐	☐
:Semi-skimmed	☐	☐	☐	☐	☐	☐
:Skimmed	☐	☐	☐	☐	☐	☐
Butter	☐	☐	☐	☐	☐	☐
Margarine (e.g., Stork, Clover)	☐	☐	☐	☐	☐	☐
Polyunsaturated margarine (e.g., Flora, sunflower)	☐	☐	☐	☐	☐	☐
Low fat spreads (e.g., Outline, Gold)	☐	☐	☐	☐	☐	☐
Ice cream	☐	☐	☐	☐	☐	☐
Yoghurt, Fromage Frais	☐	☐	☐	☐	☐	☐
Cheese (e.g., cheddar, cream cheese)	☐	☐	☐	☐	☐	☐
Low fat cheese (e.g., cottage, reduced fat cheese)	☐	☐	☐	☐	☐	☐
Eggs - fried	☐	☐	☐	☐	☐	☐
Eggs - not fried (boiled, poached, in baking)	☐	☐	☐	☐	☐	☐
Cheese and/or egg dishes	☐	☐	☐	☐	☐	☐

**MEATS AND FISH**	2 or more times a day	Every day	3-5 times a week	1-2 times a week	1-3 times a month	Rarely-never
Beef - roast/steak	☐	☐	☐	☐	☐	☐
Lamb - roast/chops	☐	☐	☐	☐	☐	☐
Pork - roast/chops	☐	☐	☐	☐	☐	☐
Chicken, turkey or other poultry	☐	☐	☐	☐	☐	☐
Bacon or gammon	☐	☐	☐	☐	☐	☐
Meat dishes (e.g., stew, curry, chilli)	☐	☐	☐	☐	☐	☐
Canned meats (e.g., corned beef, ham)	☐	☐	☐	☐	☐	☐
Sausages or beef burgers	☐	☐	☐	☐	☐	☐
Liver, kidney, paté (other offal products)	☐	☐	☐	☐	☐	☐

How often do you eat each of the following foods? (tick the appropriate box on each line)

**MEATS AND FISH ctd.**	2 or more times a day	Every day	3-5 times a week	1-2 times a week	1-3 times a month	Rarely-never
Sausages or beef burgers	☐	☐	☐	☐	☐	☐
Liver, kidney, paté (other offal products)	☐	☐	☐	☐	☐	☐
Sausages or beef burgers	☐	☐	☐	☐	☐	☐
Liver, kidney, paté (other offal products)	☐	☐	☐	☐	☐	☐
Fish and seafood - not fried	☐	☐	☐	☐	☐	☐
- fried	☐	☐	☐	☐	☐	☐
- canned (e.g., tuna)	☐	☐	☐	☐	☐	☐

**BREAD AND CEREALS**	2 or more times a day	Every day	3-5 times a week	1-2 times a week	1-3 times a month	Rarely-never
White bread	☐	☐	☐	☐	☐	☐
Brown/granary bread	☐	☐	☐	☐	☐	☐
Wholemeal bread (including chapattis)	☐	☐	☐	☐	☐	☐
Sweet biscuits (plain and chocolate)	☐	☐	☐	☐	☐	☐
Crackers/crisp bread	☐	☐	☐	☐	☐	☐
Cakes/buns/pastries	☐	☐	☐	☐	☐	☐
Puddings (e.g., fruit pies, cheesecake)	☐	☐	☐	☐	☐	☐
Breakfast cereal:						
- High fibre (e.g., bran flakes, Weetabix)	☐	☐	☐	☐	☐	☐
- Ordinary (e.g., cornflakes, rice krispies)	☐	☐	☐	☐	☐	☐
- Muesli	☐	☐	☐	☐	☐	☐
Rice or pasta	☐	☐	☐	☐	☐	☐

**FRUIT AND VEGETABLES**	2 or more times a day	Every day	3-5 times a week	1-2 times a week	1-3 times a month	Rarely-never
Apples, pears	☐	☐	☐	☐	☐	☐
Oranges, grapefruit or other citrus fruit	☐	☐	☐	☐	☐	☐
Bananas	☐	☐	☐	☐	☐	☐
Green vegetables (e.g., cabbage, peas)	☐	☐	☐	☐	☐	☐
Carrots, tomatoes (fresh or canned)	☐	☐	☐	☐	☐	☐
Other vegetables (including salad vegetables)	☐	☐	☐	☐	☐	☐
Baked beans	☐	☐	☐	☐	☐	☐
Other beans/lentils (e.g., dahl)	☐	☐	☐	☐	☐	☐
Vegetable dishes (e.g., stew, curry - NO meat)	☐	☐	☐	☐	☐	☐
Potatoes - chips, roasted, fried	☐	☐	☐	☐	☐	☐
- not fried (boiled, baked)	☐	☐	☐	☐	☐	☐

How often do you eat each of the following foods? (tick the appropriate box on each line)

	2 or more times a day	Every day	3-5 times a week	1-2 times a week	1-3 times a month	Rarely-never
**BEVERAGES**						
Beer or lager	☐	☐	☐	☐	☐	☐
Wine, sherry or spirits	☐	☐	☐	☐	☐	☐
Tea or coffee	☐	☐	☐	☐	☐	☐
Squash or fizzy drinks	☐	☐	☐	☐	☐	☐
Low calorie drinks	☐	☐	☐	☐	☐	☐
Pure fruit juices	☐	☐	☐	☐	☐	☐
**MISCELLANEOUS**						
Chocolate	☐	☐	☐	☐	☐	☐
Sweets	☐	☐	☐	☐	☐	☐
Sweet spreads (e.g., jam, marmalade)	☐	☐	☐	☐	☐	☐
Sugar (e.g., in tea/coffee, on cereal)	☐	☐	☐	☐	☐	☐
Crisps and savoury snacks	☐	☐	☐	☐	☐	☐
Nuts (including peanut butter)	☐	☐	☐	☐	☐	☐
Sauces and pickles (e.g., ketchup, Branston)	☐	☐	☐	☐	☐	☐
Salad oils, dressings, mayonnaise	☐	☐	☐	☐	☐	☐

Are there any other foods that have not been recorded that you regularly eat?
Please write what they are and how often you eat them:

	2 or more times a day	Every day	3-5 times a week	1-2 times a week	1-3 times a month	Rarely-never
______________________	☐	☐	☐	☐	☐	☐
______________________	☐	☐	☐	☐	☐	☐
______________________	☐	☐	☐	☐	☐	☐

<u>PLEASE COMPLETE THE FOLLOWING QUESTIONS ABOUT YOURSELF:</u>

8. How tall are you? ______feet ______inches **OR** ______metres

9. How much do you weigh? ______stones ______lbs **OR** ______Kgs

Please turn over

Smoking Questionnaire

You have the right to decide not to complete the questionnaire if you don't want to.

But please tick this box if you are happy to take part in this study → ☐

We'll never ask for your name when you do this questionnaire

There are no right or wrong answers ☺

Your parents and teacher won't see your results. They're private.

My 4-letter Password

First & last letters of my important female name (e.g. Sarah – SH)

☐ ☐

First & last letters of my important male name (e.g. Adnan – AN)

☐ ☐

Not sure of the **exact** names you chose, e.g. Rebecca or Becky? Andrew or Andy?
No problem ☺ Please just write down the different names they're known by here:

Please fill in the password boxes above with the **same password** you've been using for this University research.

Are you a **new student**? Please tick here ☐ I'm new

You'll need to create a 4-letter password and write it in the boxes above **plus** note it down somewhere safe.
Your teacher or University researcher will be able to help – just ask ☺ .

1. **a) I am:** ☐ a boy ☐ a girl **b) My age:** __ __ years

2. **Do you smoke cigarettes these days?** ☐ Yes ☐ No

3. **Who smokes in your family now?** Tick **all** the people who smoke at the moment:

☐ Mum (including step-mum) ☐ Dad (including step-dad) ☐ Grandma ☐ Grandad

☐ Older brother (including step-brother) ☐ Younger brother (including step-brother)

☐ Older sister (including step-sister) ☐ Younger sister (including step-sister)

☐ Other people. Who? Please list in this box→

☐ No-one smokes in my family

4. **Read the following statements carefully and tick the ONE that describes you.**

☐ I have never smoked

☐ I have only ever tried smoking once

☐ I used to smoke sometimes, but I never smoke cigarettes now

☐ I sometimes smoke cigarettes now, but I don't smoke as many as one a week

☐ I usually smoke between one and six cigarettes a week

☐ I usually smoke more than six cigarettes a week

5. **<u>ONLY</u> answer this question if you have <u>never smoked</u> or <u>only tried it once.</u>**

a) Put a tick beside the statement that best describes you:

- ☐ I have **never** had one puff of a cigarette
- ☐ I did **once** have a puff of a cigarette
- ☐ I have **tried smoking a few times**, but I never smoke now
- ☐ I do **sometimes smoke cigarettes**, but not as many as one a week

b) How many times have you been offered a cigarette?

Please answer in this box- [**times**]

c) How many times did you smoke the cigarette?

Please answer in this box- [**times**]

d) What did you usually do or say when offered a cigarette?

Please write in this box-

[   ]

6. **How many of your friends smoke**?

All of them	Most but not all	Half and half	Only a few	None of them
☐	☐	☐	☐	☐

For these next questions, tick <u>ONE box per question</u> to show what you think.

**7.** a) I am confident I could resist smoking:	**strongly disagree**	☐	☐	☐	☐	☐	**strongly agree**
b) For me to not smoke would be:	**difficult**	☐	☐	☐	☐	☐	**easy**
c) How much control do you feel you have over not smoking?	**no control**	☐	☐	☐	☐	☐	**complete control**

**8.** a) Most of my friends think...	**I should smoke**	☐	☐	☐	☐	☐	**I should not smoke**
b) My best male friend thinks...	**I should smoke**	☐	☐	☐	☐	☐	**I should not smoke**
c) My best female friend thinks...	**I should smoke**	☐	☐	☐	☐	☐	**I should not smoke**
d) My family think...	**I should smoke**	☐	☐	☐	☐	☐	**I should not smoke**
e) People who are important to me think...	**I should smoke**	☐	☐	☐	☐	☐	**I should not smoke**

**9.** a) I plan not to smoke:	**strongly disagree**	☐	☐	☐	☐	☐	**strongly agree**
b) I don't want to smoke:	**strongly disagree**	☐	☐	☐	☐	☐	**strongly agree**
c) I will try not to smoke:	**strongly disagree**	☐	☐	☐	☐	☐	**strongly agree**

**10.** a) I can say no to smoking, even at school:	**strongly disagree**	☐	☐	☐	☐	☐	**strongly agree**
b) I can say no to smoking even when I'm offered a cigarette:	**strongly disagree**	☐	☐	☐	☐	☐	**strongly agree**
c) I can say no to smoking, even if my friends want me to smoke:	**strongly disagree**	☐	☐	☐	☐	☐	**strongly agree**
d) I can say no to smoking, even if I was the only one in the group not smoking:	**strongly disagree**	☐	☐	☐	☐	☐	**strongly agree**
e) I can say no to smoking, even if I feel a bit left out of the group:	**strongly disagree**	☐	☐	☐	☐	☐	**strongly agree**
f) I can say no to smoking, even if I feel like smoking:	**strongly disagree**	☐	☐	☐	☐	☐	**strongly agree**

11. Tick one box for each question (questions a,b,c,d,e,f and g) to show what you think.

For me, smoking would be...

**a)**	**bad**	☐	☐	☐	☐	☐	**good**
**b)**	**harmful**	☐	☐	☐	☐	☐	**beneficial**
**c)**	**unpleasant**	☐	☐	☐	☐	☐	**pleasant**
**d)**	**unenjoyable**	☐	☐	☐	☐	☐	**enjoyable**
**e)**	**foolish**	☐	☐	☐	☐	☐	**wise**
**f)**	**not fun**	☐	☐	☐	☐	☐	**fun**
**g)**	**healthy**	☐	☐	☐	☐	☐	**unhealthy**

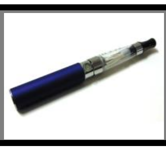

These questions ask about your experience of e-cigarettes and/or vapourisers

12. An e-cigarette or vapouriser is a tube that sometimes looks like a normal cigarette and has a glowing tip. They all puff a vapour that looks like smoke but unlike normal cigarettes, they don't burn tobacco.

Have you ever heard of e-cigarettes or vapourisers? (Please tick one box)

☐ Yes I have ☐ No I haven't ☐ I don't know

13. Which ONE of the following is closest to describing your experience of e-cigarettes or vapourisers? (Please tick one box)

☐ I have never used them

☐ I have tried them once or twice

☐ I use them sometimes (more than once a month but less than once a week)

☐ I use them often (more than once a week)

These last questions ask about how you are today 😐☹☺

Instructions

These next 9 questions ask about how you are **today**. For each question, read all the choices and decide which one is most like you **today**.

Then put a tick in the box next to it like this ☑. Only tick **one** box for each question.

Example: Today I feel quite upset so I will tick this box:

Upset

☐ I don't feel upset today
☐ I feel a little bit upset today
☐ I feel a bit upset today
☑ I feel quite upset today
☐ I feel very upset today

Now think about and answer the rest of the questions over the page

1. Worried

☐ I don't feel worried today
☐ I feel a little bit worried today
☐ I feel a bit worried today
☐ I feel quite worried today
☐ I feel very worried today

2. Sad

☐ I don't feel sad today
☐ I feel a little bit sad today
☐ I feel a bit sad today
☐ I feel quite sad today
☐ I feel very sad today

3. Pain

☐ I don't have any pain today
☐ I have a little bit of pain today
☐ I have a bit of pain today
☐ I have quite a lot of pain today
☐ I have a lot of pain today

4. Tired

☐ I don't feel tired today
☐ I feel a little bit tired today
☐ I feel a bit tired today
☐ I feel quite tired today
☐ I feel very tired today

5. Annoyed

☐ I don't feel annoyed today
☐ I feel a little annoyed today
☐ I feel a bit annoyed today
☐ I feel quite annoyed today
☐ I feel very annoyed today

6. School Work/Homework (such as reading, writing, doing lessons)

☐ I have no problems with my schoolwork/homework today
☐ I have a few problems with my schoolwork/homework today
☐ I have some problems with my schoolwork/homework today
☐ I have many problems with my schoolwork/homework today
☐ I can't do my schoolwork/homework today

7. Sleep

☐ Last night I had no problems sleeping
☐ Last night I had a few problems sleeping
☐ Last night I had some problems sleeping
☐ Last night I had many problems sleeping
☐ Last night I couldn't sleep at all

8. Daily routine (things like eating, having a bath/shower, getting dressed)

☐ I have no problems with my daily routine today
☐ I have a few problems with my daily routine today
☐ I have some problems with my daily routine today
☐ I have many problems with my daily routine today
☐ I can't do my daily routine today

9. Able to join in activities (things like playing out with your friends, doing sports, joining in things)

☐ I can join in any activities today
☐ I can join in with most activities today
☐ I can join in with some activities today
☐ I can join in with a few activities today
☐ I can join in with no activities today

Thank you very much for filling this in for us. Was there anything you didn't understand? Please tell us:

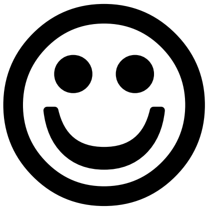

Study 3: T1 and T2 questionnaires presented online.

Description of Study on Prolific Academic Website

Physical Activity Study – Two part

This is a two part study that will ask you to answer some questions about physical activity over the next month.

The first part of the study will take approximately 8 minutes to complete.

After one month, you will be invited via email to take part in the second part of the study. This second questionnaire will take approximately 5 minutes to complete.

Please only complete the first survey if you are happy to also complete the second survey. Both parts combined will give you a total of £2.50 after completion.

The study has been approved by the University of   , School of Psychology Ethics Committee (Ref: XXX, Date XXX).

Participant Information Sheet and Consent Form (Each Questionnaire)

You are being invited to take part in the 'Behaviour Study' conducted by researchers at the University of Leeds. This is a two part study and in this study, we are particularly interested in investigating the factors that influence your participation in physical activity over the next month.

The first session of this study will take approximately 8 minutes to complete. First you will be asked to provide some basic demographic details (e.g., age, gender, education, employment status), followed by some questions on your thoughts towards performing physical activity over the next month and some more general questions about your thoughts and feelings.

One month after you complete the first questionnaire, you will be emailed via Prolific Academic to invite you to complete another short questionnaire which will take approximately 5 minutes to complete.

All your information will be kept confidential, and we will never ask you to provide you with personal information such as name or email address. We will not be able to see this personal information on the Prolific Academic website either.

Please make sure that you fill in your Prolific Academic ID correctly, as we will use this to invite you to participate in the second part of the study, and will also use it to ensure that you receive the payment for your participation.

Although we would really appreciate it if you would complete both parts of the study, you are free to withdraw from the study at any time by not completing either questionnaire.

Once you have completed the second questionnaire, you will be debriefed about the aims of the study.

If you have any questions at any time, feel free to contact the research team (insert study email address) or the Principal Investigator).

It is your decision whether you want to take part in the study, by clicking continue at the bottom of this page, you are indicating your consent to take part. However, you will still be free to withdraw at any time without giving a reason. All the data collected from you during the experiment will be kept completely confidential, and you will not be able to be identified in any reports or publications of the findings.

Consent Declaration

I confirm that I have read and understand the information sheet explaining the above research project and I have had the opportunity to contact the researchers about the project if I have any questions.

☐ I agree

I understand that I remain free to withdraw from the study at any time without giving a reason and without there being any negative consequences.

☐ I agree

I understand that once my responses to either questionnaire are submitted, my responses will be anonymised and my data cannot be withdrawn.

☐ I agree

I agree for the data collected from me to be used in further research.

☐ I agree

I understand that I will not be able to be identified by any reports or publications as all data is anonymous and I will be identified only by a code.

☐ I agree

I agree to participate in this research (questionnaires at time 1 and 2)

☐ I agree

This research has received ethical approval by the University of , School of Psychology Ethics Committee (ref:xxx-xx, date: xx/xx/xx).

Debrief Sheet

Thank you for taking part in the behaviour study. This debrief sheet will provide you with more information about the aims of the research.

In this study, we tested:

1) Whether different patterns of attitudes were most strongly related to engagement with physical activity over a one month period.

2) Whether thoughts or feelings were more or less important in driving physical activity in those with different views (e.g., had consistent or mixed attitudes to physical activity).

The following website provides details of key health behaviours and their links to health outcomes:

https://www.nhs.uk/news/food-and-diet/healthy-behaviours-extend-life/

If you have any other questions, please contact the research team on [insert study email address], or the Principal Investigator Professor).

This research has received ethical approval by the University of , School of Psychology Ethics Committee (ref:xxx-xx, date: xx/xx/xx).

Questionnaires

Time 1 Questionnaire

Demographics

1. Age
2. Gender
3. Nationality
4. Ethnicity
5. Highest Education level
6. Employment status
7. SES (Income, SES ladders)
8. Number of people in household
9. Postcode/Zipcode

The next set of questions refer to **engaging in the recommended levels of physical activity each week**. This involves engaging in a minimum of 150 minutes of moderate activity (e.g. cycling or fast walking) or 75 minutes of vigorous activity (e.g. running), or a mixture of vigorous and moderate activity every week plus strength exercises on two or more days a week that work all the major muscles (legs, hips, back, abdomen, chest, shoulders and arms).

10. My **engaging in the recommended levels of physical activity each week** over the next month would be: (1 not at all – 7 extremely)

(Evaluative)

Bad
Good
Negative
positive
unfavourable
Favourable
Unimportant
Important

(Affective)

Unenjoyable
Enjoyable
Unpleasurable
Pleasurable
Boring
Exciting
Disagreeable
Agreeable

(Cognitive)

Useless
Useful
Harmful
Beneficial
Unhealthy
Healthy
Worthless
valuable

11. I plan to **engage in the recommended levels of physical activity each week** over the next month (1 Strongly disagree – 7 Strongly agree).
12. I intend to **engage in the recommended levels of physical activity each week** over the next month (1 Strongly disagree – 7 Strongly agree)
13. I will make an effort to **engage in the recommended levels of physical activity each week** over the next month (1 Strongly disagree – 7 Strongly agree).
14. I will definitely try to **engage in the recommended levels of physical activity each week** over the next month (1 Strongly disagree – 7 Strongly agree).
15. I am going to **engage in the recommended levels of physical activity each week** over the next month (1 Strongly disagree – 7 Strongly agree).

16. In relation to my **engaging in the recommended levels of physical activity each week** I feel…..

No conflict 1 2 3 4 5 6 7 8 9 Maximum conflict

17. In relation to my **engaging in the recommended levels of physical activity each week** I feel…..

No indecision 1 2 3 4 5 6 7 8 9 Maximum indecision

18. In relation to my **engaging in the recommended levels of physical activity each week** I feel…..

Clear reactions 1 2 3 4 5 6 7 8 9 Mixed reactions

19. Over the past month, how many weeks did you **engage in the recommended levels of physical activity?** (____ weeks)
20. How frequently did you **engage in the recommended levels of physical activity each week** over the last month? (1 never – 7 always)
21. Over the last month, **I engaged in the recommended levels of physical activity each week** (1 never – 7 always)
22. Over the last month, how many minutes of **moderate activity** did you engage in each week? ___ minutes per week.
23. Over the last month, how many minutes of **vigorous activity** did you engage in each week? ___ minutes per week.
24. Over the last month, how days did you **engage in strength exercises** that work all the major muscles? ___ days over month.

25. I want to **engage in the recommended levels of physical activity each week** over the next month (1 Strongly disagree – 7 Strongly agree).
26. **Engaging in the recommended levels of physical activity each week** over the next month is something I really desire to do (1 Strongly disagree – 7 Strongly agree).

27. I desire to **engage in the recommended levels of physical activity each week** over the next month (1 Strongly disagree – 7 Strongly agree).
28. **Engaging in the recommended levels of physical activity each week** over the next month is something I really do not want to do (1 Strongly disagree – 7 Strongly agree).
29. I feel I should **engage in the recommended levels of physical activity each week** over the next month (1 Strongly disagree – 7 Strongly agree).
30. I feel I ought to **engage in the recommended levels of physical activity each week** over the next month (1 Strongly disagree – 7 Strongly agree).
31. **Engaging in the recommended levels of physical activity each week** over the next month is not something I do (1 Strongly disagree – 7 Strongly agree).
32. **Engaging in the recommended levels of physical activity each week** over the next month is something I … (1 Ought not to do – 7 Ought to do).
33. To what extent do you feel you need to **engage in the recommended levels of physical activity each week** over the next month (1 Not at all – 7 An extremely great extent).

34. I would prioritize **engaging in the recommended levels of physical activity each week** over other goals important to me over the next month (1 Strongly disagree – 7 Strongly agree).
35. For me, ensuring I **engage in the recommended levels of physical activity each week** over the next month is a… (1 Low priority – 7 High priority).
36. **Engaging in the recommended levels of physical activity each week** over the next month is not a high priority for me (1 Strongly disagree – 7 Strongly agree).
37. When I think of things I want to achieve, e**ngaging in the recommended levels of physical activity each week** over the next month is near the top of my list (1 Strongly disagree – 7 Strongly agree).

38. If I did not **engage in the recommended levels of physical activity each week** over the next month I would regret it (1 Strongly disagree – 5 Strongly agree).
39. If I did not **engage in the recommended levels of physical activity each week** over the next month I would feel guilty (1 Strongly disagree – 5 Strongly agree).
40. If I did not **engage in the recommended levels of physical activity each week** over the next month I would feel sad (1 Strongly disagree – 5 Strongly agree).
41. If I did not **engage in the recommended levels of physical activity each week** over the next month I would feel disappointed(1 Strongly disagree – 5 Strongly agree).
42. If I did **engage in the recommended levels of physical activity each week** over the next month I would feel proud (1 Strongly disagree – 5 Strongly agree).
43. If I did **engage in the recommended levels of physical activity each week** over the next month I would feel joy (1 Strongly disagree – 5 Strongly agree).
44. If I did **engage in the recommended levels of physical activity each week** over the next month I would feel happy (1 Strongly disagree – 5 Strongly agree).
45. If I did **engage in the recommended levels of physical activity each week** over the next month I would feel satisfied (1 Strongly disagree – 5 Strongly agree).

Time 2 Questionnaire

The next set of questions refer to **engaging in the recommended levels of physical activity each week**. This involves engaging in a minimum of 150 minutes of moderate activity (e.g. cycling or fast walking) or 75 minutes of vigorous activity (e.g. running), or a mixture of vigorous and moderate activity every week plus strength exercises on two or more days a week that work all the major muscles (legs, hips, back, abdomen, chest, shoulders and arms).

1. My **engaging in the recommended levels of physical activity each week** over the next month would be: (1 not at all – 7 extremely)

(Evaluative)

Bad
Good
Negative
positive
unfavourable
Favourable
Unimportant
Important

(Affective)

Unenjoyable
Enjoyable
Unpleasurable
Pleasurable
Boring
Exciting
Disagreeable
Agreeable

(Cognitive)

Useless
Useful
Harmful
Beneficial
Unhealthy
Healthy
Worthless
valuable

2. I plan to **engage in the recommended levels of physical activity each week** over the next month (1 Strongly disagree – 7 Strongly agree).
3. I intend to **engage in the recommended levels of physical activity each week** over the next month (1 Strongly disagree – 7 Strongly agree)
4. I will make an effort to **engage in the recommended levels of physical activity each week** over the next month (1 Strongly disagree – 7 Strongly agree).
5. I will definitely try to **engage in the recommended levels of physical activity each week** over the next month (1 Strongly disagree – 7 Strongly agree).
6. I am going to **engage in the recommended levels of physical activity each week** over the next month (1 Strongly disagree – 7 Strongly agree).

7. In relation to my **engaging in the recommended levels of physical activity each week** I feel.....

No conflict 1 2 3 4 5 6 7 8 9 Maximum conflict

8. In relation to my **engaging in the recommended levels of physical activity each week** I feel.....

No indecision 1 2 3 4 5 6 7 8 9 Maximum indecision

9. In relation to my **engaging in the recommended levels of physical activity each week** I feel…..

Clear reactions 1 2 3 4 5 6 7 8 9 Mixed reactions

10. Over the past month, how many weeks did you **engage in the recommended levels of physical activity?** (____ weeks)
11. How frequently did you **engage in the recommended levels of physical activity each week** over the last month? (1 never – 7 always)
12. Over the last month, **I engaged in the recommended levels of physical activity each week** (1 never – 7 always)
13. Over the last month, how many minutes of **moderate activity** did you engage in each week? ___ minutes per week.
14. Over the last month, how many minutes of **vigorous activity** did you engage in each week? ___ minutes per week.
15. Over the last month, how days did you **engage in strength exercises** that work all the major muscles? ___ days over month.

16. I want to **engage in the recommended levels of physical activity each week** over the next month (1 Strongly disagree – 7 Strongly agree).
17. **Engaging in the recommended levels of physical activity each week** over the next month is something I really desire to do (1 Strongly disagree – 7 Strongly agree).
18. I desire to **engage in the recommended levels of physical activity each week** over the next month (1 Strongly disagree – 7 Strongly agree).
19. **Engaging in the recommended levels of physical activity each week** over the next month is something I really do not want to do (1 Strongly disagree – 7 Strongly agree).
20. I feel I should **engage in the recommended levels of physical activity each week** over the next month (1 Strongly disagree – 7 Strongly agree).
21. I feel I ought to **engage in the recommended levels of physical activity each week** over the next month (1 Strongly disagree – 7 Strongly agree).
22. **Engaging in the recommended levels of physical activity each week** over the next month is not something I do (1 Strongly disagree – 7 Strongly agree).
23. **Engaging in the recommended levels of physical activity each week** over the next month is something I … (1 Ought not to do – 7 Ought to do).
24. To what extent do you feel you need to **engage in the recommended levels of physical activity each week** over the next month (1 Not at all – 7 An extremely great extent).
